# Database of literature derived cellular measurements from the murine basal ganglia

**DOI:** 10.1038/s41597-020-0550-3

**Published:** 2020-07-06

**Authors:** Ingvild E. Bjerke, Maja A. Puchades, Jan G. Bjaalie, Trygve B. Leergaard

**Affiliations:** Department of Molecular Medicine, Institute of Basic Medical Sciences, University of Oslo, Oslo, Norway

**Keywords:** Brain, Cellular neuroscience

## Abstract

Quantitative measurements and descriptive statistics of different cellular elements in the brain are typically published in journal articles as text, tables, and example figures, and represent an important basis for the creation of biologically constrained computational models, design of intervention studies, and comparison of subject groups. Such data can be challenging to extract from publications and difficult to normalise and compare across studies, and few studies have so far attempted to integrate quantitative information available in journal articles. We here present a database of quantitative information about cellular parameters in the frequently studied murine basal ganglia. The database holds a curated and normalised selection of currently available data collected from the literature and public repositories, providing the most comprehensive collection of quantitative neuroanatomical data from the basal ganglia to date. The database is shared as a downloadable resource from the EBRAINS Knowledge Graph (https://kg.ebrains.eu), together with a workflow that allows interested researchers to update and expand the database with data from future reports.

## Abstract (summary)

**Table:** 

Measurement(s)	Cell number • synapse • dendritic spine • Cell Density • Synapse density • dendritic spine density • Distribution • neuron morphology trait • basal ganglion morphology trait
Technology Type(s)	digital curation
Sample Characteristic - Organism	Mus musculus • Rattus norvegicus

Machine-accessible metadata file describing the reported data: 10.6084/m9.figshare.12199088

## Background & Summary

Quantitative knowledge about the number and normal variation of different cell types of the brain and their subcellular elements, such as synapses and dendritic spines, is of broad interest for neuroscientists. This is important for several purposes, including building and constraining computational models^1–3^, guiding new experimental research, and comparing data from individual or groups of subjects. The need for quantitative measurements of neural architecture has led to development of numerous experimental methods for unbiased quantification of neuroanatomical features. Examples include cell counting methods^4^, stereological approaches to obtain numbers, areas or volumes^5,6^, and point pattern analyses to characterise spatial distributions of cells or cellular elements^7,8^. The results of such studies are typically published in original papers, reporting e.g. estimates of total numbers or densities of cells^9,10^ or relative amounts of cells, synapses, spines, or other parameters in different experimental groups^11,12^. While individual papers may be easily interpreted, it is becoming increasingly challenging to overview the steadily growing amount of publications^13^ and to evaluate the consistency and comparability of information. The traditional research paper format is not particularly well suited to make comparisons, as data may be distributed across text, tables and figures, with units of measurements and nomenclatures that vary across papers. Although units of measurements can be effectively converted and nomenclature differences may be possible to resolve, this requires significant time and effort from the reader. In some cases, findings are reported in non-standard units (e.g. as percentage of control, number per section), which may make them impossible to compare to other results. Researchers investigating brain structure and function in animal models may find it difficult to answer relatively simple questions, such as: What is the average number of cells or subcellular structures in my brain region of interest, and how much do these numbers vary? Which parameters have been quantified before and what were the methods used to do so? Can data from two studies be compared? Are the results reported in the literature within the same range?

Neuroanatomical information is available from several databases. The temporal-lobe database (www.temporal-lobe.com) presents connections in the rat hippocampal region are presented schematically in an interactive PDF, allowing the user to quickly view aggregated information^14^. In the Brain Architecture Management System (BAMS, https://bams1.org/) project, Bota and colleagues compiled reports and scored the strength of connections between regions across the brain on a semi-quantitative scale^15^. The hippocampome (www.hippocampome.org), a database of neuronal cell types in the hippocampus, contains interactive matrices showing the location, cytochemistry, electrophysiology, and connectivity of different cell types^16^. The NeuroMorpho database^17^ (www.neuromorpho.org) is an extensive collection of published neuron morphologies, with useful quantitative information about the features of individual neurons. In addition, several efforts have been made to estimate brain cell numbers in histological material using computational image analysis^18–20^. To our knowledge, no systematic effort has been made to collect and normalise information from several sources about the number and distribution of cells, synapses and spines in different brain regions in a database.

We here present a database of publicly available quantitative measurements of cells, synapses and dendritic spines of the frequently investigated murine basal ganglia. These are regions of high interest for basic experimental studies of voluntary movements, procedural learning and neurodegenerative diseases such as Parkinson’s and Huntington’s disease^21,22^. Quantitative information about the cellular architecture of these regions in normal animals is needed for computational modelling efforts^1,2^, and represent an important benchmark for interpretation of results from experimental studies in different animal disease models^23,24^. The database holds > 1200 quantitative estimates derived from the literature and public repositories, normalised to standard units of measurements and mapped to common anatomical reference atlases. To our knowledge, this is the most extensive collection of available information on cellular basal ganglia parameters to date. The database is publicly shared via EBRAINS^25^, together with a workflow for updating it with results from future analyses. We believe this can be a valuable benchmark resource for anatomical studies or efforts to model the murine basal ganglia.

## Methods

### Overview of study design

We created a database of data derived from the literature and public repositories. We here use the term derived data to describe the specific analytic results of a study, e.g. the number of cells in a given region, as opposed to the raw data that were used to generate this number. We limited our scope to quantitative information about number, distribution and morphology of cells and subcellular elements of the normal, rodent basal ganglia. We here consider the concept of the basal ganglia to include dorsal and ventral parts of the striatum (caudoputamen and nucleus accumbens) and pallidum (external globus pallidus, entopeduncular nucleus and ventral pallidum), as well as the subthalamic nucleus and substantia nigra^26,27^. We designed a database using Microsoft Access, and set up a search string to query the literature. Specific inclusion criteria were used to narrow the number of papers to include. For each paper, the methods and results sections were carefully read and annotated, and relevant metadata elements were integrated in the database. We also searched for repositories with relevant data. Wherever necessary and possible, we standardised terms and units used to describe data, and novel workflows were developed to map data to common schemes for regions and cell types of interest. Lastly, in order to make our database accessible and usable to the community, we shared it as a dataset through the EBRAINS Knowledge Graph (RRID:SCR_017612). Each main part of the study design (database design, search strategy, data / metadata standardisation, and data sharing) will be elaborated in the following.

### Database design

**Fig. 1:**
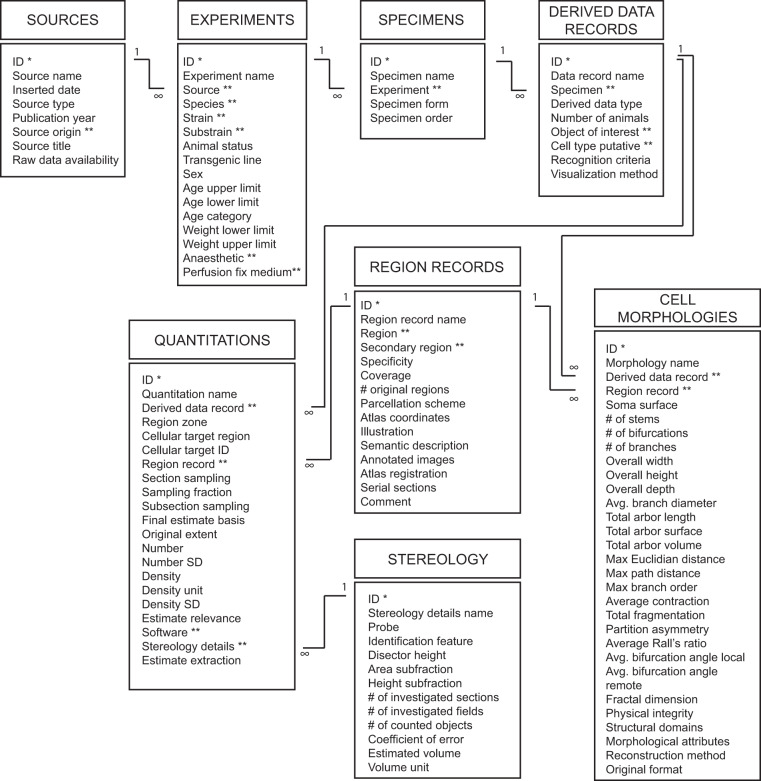
Key tables in the database. Information about each source (publication or repository) is stored in the “Sources” table. Each source may have one or more related record in the “Experiments” table. An experiment may have one or more specimens, information about which is stored in the “Specimens” table. Specimens are not defined at the level of individual animals, but rather at the level of different *types* of specimen, e.g. a brain, serial sections, etc. The specimens are organised in levels, i.e. the whole brain is the primary specimen, and if a series of sections are cut from that brain it would be a secondary specimen. A specimen may have one or more derived data records, information about which is stored in the derived data records table. A derived data record can relate to one or more records in either the “Quantitations” or “Cell morphologies” table. A record in the quantitations table may or may not have a related record in the “Stereology details” table, depending on whether such procedures have been used; if similar parameters were used for stereological counting, several quantitations may relate to the same record in this table. The “Region records” table stores information about the location of one or more quantitation or morphology in standard EBRAINS atlas terms; in addition, information is stored about the accuracy of the translation from the original term used by the authors, as well as the documentation provided to support location information. For more information about each field in all tables of the database, including those not shown in this figure, see Supplementary File 1. *Primary key; **Foreign key.

We organised data derived from the literature in a Microsoft Access database with 45 tables, the most important of which are summarised in Fig. 1. All fields in all tables of the database are listed and explained in Supplementary File 1.

### Search strategy

#### Literature search strategy

PubMed was queried via Ovid Medline for papers from 1946-present. This search string included key words related to (1) species of interest, i.e. rat and mouse; (2) brain regions of interest, i.e. basal ganglia regions; (3) methods of interest, i.e. typical methods employed in anatomical and morphological studies; and (4) parameters of interest, i.e. numbers, densities or distributions of cells, synapses, axonal boutons, or dendritic spines. The papers needed to contain one key word from each of the categories in either the title or the abstract (the parts were combined with AND operators).

Three searches were performed, and search strings used are included in Supplementary File 2. In this iteration, we included all number, density and distribution data from the basal ganglia of adult, naïve rats or mice. However, the derived data was quite heterogeneous and few numbers could be compared. In the second and third search, we opted to include more data representing similar parameters. To this end, we narrowed the scope to data from the substantia nigra (second search) and caudoputamen (third search), but broadened the inclusion criteria to include all control animals of all (postnatal) ages.

The first search was performed on January 3^rd^, 2018, and a total of 2246 papers were returned. All of these papers were manually screened, and included or excluded based on a set of predefined criteria. The data had to fit with the overall criteria specified in the search string (e.g. neuroscience related, murine data, and original article format) and be available in English. Furthermore, we only included papers with data related to adult, *naïve* animals, that is, animals that had not been subject to any experimental or control manipulations, behavioural tests or training, or any other experimental intervention. The only exception to this criterion was made where pooled data from two control groups were given (e.g. sham operation and naïve control) where individual measurements had been statistically compared and proven similar. Animals with genetic manipulations, e.g. fluorescent expression in certain cells, were also excluded. Non-naïve animals were excluded to reduce the number of included publications in this first iteration of the search. However, in later and more specific queries, studies of non-naïve animals were included in order to avoid missing clearly relevant data (see below). Papers had to present *quantitative* data of interest in text or tabular format, excluding papers presenting data in graphs only. Lastly, data needed to be possible to normalise to a common unit of measurement. This generally meant that data had to be presented as numbers representing either a region of interest or a standard unit (square or cubic nano, micro-, or millimetre). In contrast, we excluded data that were presented as relative measures such as percentage of control or numbers per section. After manual screening, we included 65 publications with data of interest from the normal adult rat or mouse basal ganglia. An additional eight papers were included through tracking references of particularly relevant papers approach so that 72 papers were ultimately included in the first search.

**Fig. 2:**
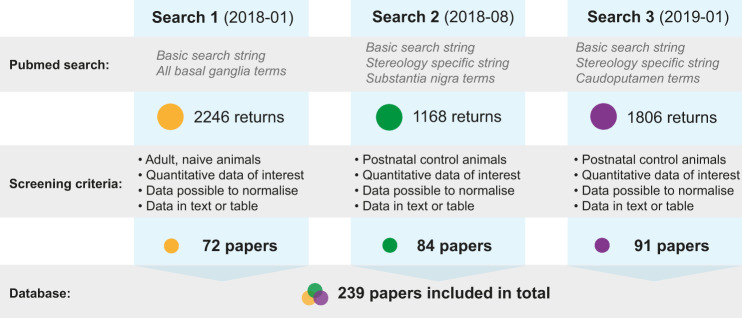
Search strategy, inclusion criteria and results for the literature search. We performed three iterations of our PubMed search (search 1–3) and manually screened all returned papers to collect data of interest for the current project. The basic search string was the same in each of these iterations, but with changes in which basal ganglia regions were included. In the second and third search, we also used an additional, targeted search string to retrieve sterological studies. The full search strings are included in Supplementary file 2. In the first iteration of the search, we only included data from naïve (untreated) adult animals from all basal ganglia regions. In the second and third search, we narrowed our scope to the substantia nigra and caudoputamen, respectively, but included data from all (postnatal) control animals. See text for further details about the criteria. In the end, 239 unique papers were included in our database (some papers appeared in two or more of the searches).

The search string employed was a compromise between sensitivity and specificity, with the use of keywords related to tissue preparation method (e.g. immunohistochemistry, immunofluorescence, histology) reducing the number of search entries considerably. Since a substantial proportion of papers found with the initial search were excluded during manual screening, these keywords were included to narrow the number of papers returned. Nevertheless, to avoid missing clearly relevant papers, we performed an additional, targeted search for papers particularly conducting stereological counting in the next iterations of the search. Thus, two separate search strings were used for the second search: 1) the same string as in the first search, but with substantia nigra keywords only (performed on August 14^th^, 2018); and 2) an additional search string including only (stereolog*) and the keywords related to substantia nigra (performed on August 22^nd^, 2018). In the third search, we repeated both parts of the second search, but with the striatum (caudoputamen) as the region of interest. The two parts of the last search were performed 1) on November 30^th^, 2018 and 2) on January 17^th^, 2019. All the papers were screened manually, according to essentially the same criteria as for the first search, except that we included all control animals of all (postnatal) ages, including those genetically altered to express fluorescence in certain cells. We also included studies using animals that had been treated according to standard neuroanatomical protocols, e.g. axonal tract tracing experiments. The second search returned 1168 papers of which 84 were included, while the third search yielded 1806 papers of which 91 were included. Because some of the papers appeared in more than one of the search rounds, the total number of publications ultimately included in the database was 239. The search strategy, inclusion criteria and results for each iteration of the search is summarised in Fig. 2.

To limit the selection of papers and thus the scope of the survey, we excluded papers presenting data in graphs only. However, for studies presenting some material in text and some in graphs, we digitised graphs to extract all relevant data from the paper. We used a web-based plot digitiser (https://apps.automeris.io/wpd/) to import graph images, added reference points, and extracted the relevant means and error measurements. As this approach was quite time consuming, we used it for selected papers in the first and second search only. We included a field in the database to specify whether an estimate was extracted from text or from a graph.

#### Repository search strategy

Several data and metadata repositories exist with various types of neuroscience information, and the Neuroscience Information Framework (NIF, www.neuinfo.org; RRID:SCR_002894)^28^ catalogues these resources. We therefore searched the NIF for portals or databases related to rat or mouse, which returned 281 public repositories with information from rats or mice. From these, we selected nine resources that appeared to be relevant to the current project. To be included, a repository had to include relevant derived data in addition to appropriate metadata. Two repositories fulfilled these criteria: Neuromorpho (www.neuromorpho.org; RRID:SCR_002145; neuronal morphology information^29^, e.g. soma size, number of bifurcations; see frequently asked questions at www.neuromorpho.org for a full list) and Mouse Brain Architecture (www.brainarchitecture.org; RRID:SCR_004683; cell densities). Since the NeuroMorpho database is organised in several archives, each containing data from one laboratory, each such archive was treated as a separate source in our database, with the prefix “NMO” in the source name indicating that the data came from NeuroMorpho. A table of the evaluated repositories is included in Supplementary File 3. Lastly, we included information extracted from an Allen mouse brain *in situ* hybridisation experiment, available as a derived dataset via the EBRAINS Knowledge Graph^30^.

### Data and metadata standardisation

To give a unified view of data, we mapped them to key features in common schemes. Two particularly important such features in neuroscience are anatomical region and cell type of interest. Indeed, other databases have generally been structured around regions^14^ or cell types^16,31^, or both^15^. In the following, we describe the workflows established in this project to map data to common terms for regions and cell types of interest, as well as how all data were standardised to common units of measurement.

#### Mapping data to semantically defined anatomical regions of interest

Reference atlases are commonly used in neuroscience in order to relate data to anatomical locations in the brain; however, there are several alternatives to reference atlas available just for the rat^32–35^ or mouse brain^36,37^, that vary with respect to how they name and define regions. Data related to a specific region in one atlas are therefore not necessarily easily compared to data related to a similarly named region in another. Even data related to the same region in different versions of the same atlas may not be directly comparable, since some borders may have been significantly revised between atlas versions.

**Fig. 3:**
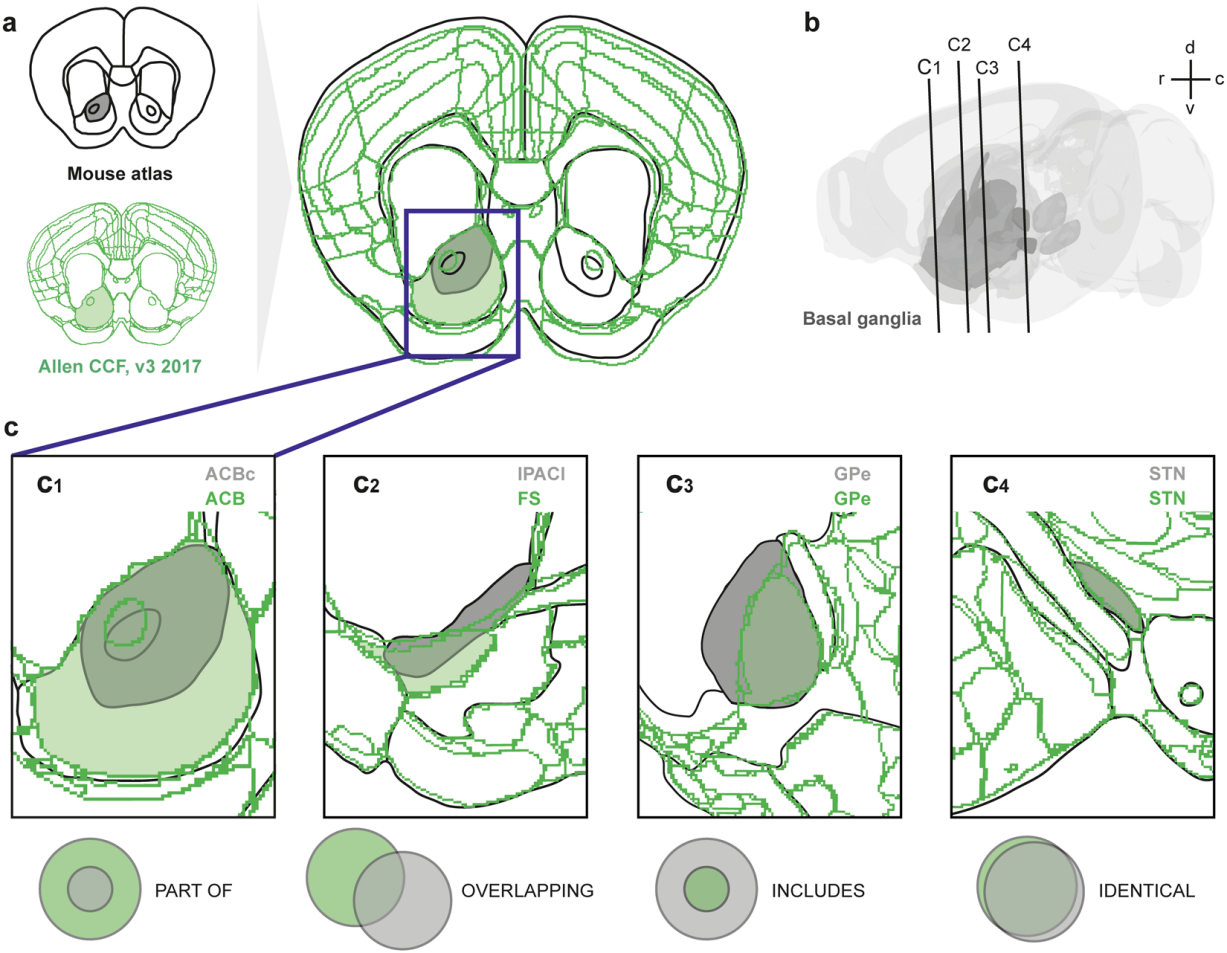
Defining topological relationships of corresponding regions in different atlases. (**a**) Comparison of 2D coronal plates taken at the level of the genu of the corpus callosum in a fictive mouse atlas (black; drawn here for illustration purposes) to spatially matching custom plates through the 3D Allen Institute Common Coordinate Framework of the mouse brain (CCF, green^37^;). By superimposing reference atlas plates with custom plates from the 3D atlas, it becomes possible to compare boundaries of regions in the two atlases. (**b**) The location of the plates shown in (**a**) and (**c**) is indicated in a transparent 3D rendering of the CCF atlas made using the Scalable Brain Atlas (https://scalablebrainatlas.incf.org/composer/?template=ABA_v3), with the basal ganglia shown in dark grey. After comparison of the co-registered atlas plates, the relationship between atlas regions is categorised as one of the types illustrated in (**a**), in which regions from the fictive atlas are shown in grey and CCF regions in green. A region is part of another region (c1), if its area is fully contained within the area of another region. For example, the fictive atlas has a region “nucleus accumbens core”, which is completely part of the larger nucleus accumbens in the CCF. Overlapping regions (c2), pertains to the situation that corresponding regions in the two compared atlases partly occupy the same space, and partly not, as seen for the lateral interstitial nucleus of the posterior limb of the anterior commissure (IPACl) in the fictive atlas, which in some parts overlaps with the fundus of striatum (FS) and caudoputamen as defined in the CCF. A region includes another region (c3), if its area fully contains the area of the other region (c3), exemplified here by two versions of the external globus pallidus (GPe), which is larger in the fictive atlas than in the CCF. Identical regions are largely similar, with little or no areas of non-overlap (c4), exemplified here with the subthalamic nucleus (STN). Relationships are exemplified here for single sections, but were determined by comparison of co-registered atlas diagrams across entire regions. Abbreviations: ACBC, nucleus accumbens, core region; ACB, nucleus accumbens; FS, fundus of striatum; GPe, globus pallidus external segment; IPACl, lateral interstitial nucleus of the posterior limb of the anterior commissure; STN, subthalamic nucleus.

In our database, all data were related to the three-dimensional (3D) standard atlas templates used in EBRAINS – the Waxholm space atlas of the rat brain (WHS, version 1.01^34,38^; RRID:SCR_017124) and the Allen Mouse Brain Common Coordinate Framework (CCF, version 3^37^). Using the QuickNII software for registration of 2D section images to 3D atlases^39^ (RRID:SCR_016854), we mapped plates from several of the most common atlases^32,33,40–49^ to WHS or CCF (Fig. 3a). The location metadata, specifying the parameters used to spatially register the different atlases to the WHS or CCF, are available as datasets from the EBRAINS Knowledge Graph^50–62^. We used the spatially co-registered atlas diagrams to inspect and define the spatial relationships between our regions of interest (basal ganglia regions) in the WHS or CCF and regions defined in other atlases. The type of relationships were categorized as *identical*, *part of, includes*, *overlapping*, or *non-overlapping* (Fig. 3c). The latter was used only in cases where regions could be expected to be related (e.g. by sharing the same name), but were found not to be. Descriptive comments about the relationships were added. In addition, to semi-quantitatively describe the degree of comparability of two regions, we applied a region comparability score, ranging from zero (non-overlapping structures) to 10 (completely identical structures). The criteria underlying this scoring system and categorization of relationships are provided in Supplementary File 4. The accumulated information about the spatial relationships defined between atlas regions are shared through the EBRAINS Knowledge Graph as separate data sets^61,62^.

The locations of data presented in papers were not always defined with use of terms from a specific reference atlas. We considered data to be related to a region in an atlas only in cases where it could be clearly inferred which region (or set of regions) in the cited atlas authors referred to. This generally involved use of a specific reference to an atlas and a region name existing in that particular atlas, with a few exceptions where authors referred to a region at a lower granularity than given in the atlas. For example, although the exact term ‘substantia nigra’ does not appear in most atlases (the region is usually subdivided, at least into a reticular and a compact part), it is reasonable to use this term to refer to the various substructures together. The crucial point is to define the inclusion or exclusion of subdivisions *as they are named in the particular atlas*. In cases where this was not clearly defined, it was reflected in our translation by storing the coverage and specificity as “unknown”. For data not defined in terms of a reference atlas, we considered the region to be defined in a ‘custom’ parcellation scheme. In these cases, knowledge about relations to our atlases could only be inferred from the documentation provided by the authors. To translate such custom terms, we therefore carefully considered the documentation and assigned an atlas term based on our knowledge about the basal ganglia regions and terms typically used to describe them. In general, more well-documented regions of interest allowed for more accurate translation with higher confidence.

For each mention of a region of interest, we included metadata describing how it was documented and stored this in the “Region records” table. We furthermore calculated a score to capture the degree to which each region of interest was documented (referred to as a “documentation score”. Different types of documentation were weighed differently, and a score between 1 and 10 was calculated. Information about the documentation factors and their weight in the documentation score can be found under the “Region records” table section in Supplementary File 1.

#### Mapping data to cell types of interest

All of the objects for which we collected quantitative information in this study belong to a cell: subcellular objects originate from a cell of interest, and reconstructed and counted cells have an identity. Cell type classification is not trivial^63,64^, as there are many complementary approaches to the task (e.g. cytochemical, electrophysiological, morphological), and thus no standard ontologies of cell types exist. To map data to cell types, we captured information about the various phenotypes that a cell might have. This approach was inspired by ongoing work from the INCF special interest group on Neuroinformatics for cell types (https://www.incf.org/sig/neuroinformatics-cell-types). We included seven broad phenotype categories: *brain region* (e.g. striatum, substantia nigra), *expression* (e.g. parvalbumin, tyrosine hydroxylase), *electrophysiology* (e.g. fast spiking), *morphology* (e.g. spiny neuron, giant neuron), *connectivity* (e.g. direct pathway neuron), *local connectivity* (e.g. perisomatic neuron), and *circuit function* (e.g. inhibitory neuron). For every derived data set, information was stored about the phenotype recorded for the particular cell. One or more phenotypes might be used in a particular study to classify the neuron type(s), and based on the phenotype(s) identified, a putative cell type was assigned. Some data spanned several different cell types, for example when numbers of *all* objects of interest were counted regardless of type (e.g. counting all dendritic spines or cell bodies in a certain area). In these cases, data are relevant for *all* cell types, and have simply been linked to the type “Cell”, “Neuron”, or “Glia”, depending on the phenotypes identified.

#### Standardisation to common units of measurement

Prior to data entry, we converted all density units to square or cubic milli- or micrometres. Standard errors were converted to standard deviations by dividing by the square root of the sample size. Information about calculations performed to standardise a measurement was entered in the database. For data given per square milli- or micrometres, we calculated the volumetric density by dividing numbers by section thickness^65^. These were entered in the database *in addition* to original 2D counts. Numbers obtained by direct counts without any corrections were corrected using Abercrombie’s formula^4,66^ prior to calculating volumetric density. These calculations are elaborated in Supplementary File 5. We did not standardise total number estimates before entering these to the database, but rather indicated whether counts were uni- or bilateral. When it was not clear whether estimates were uni- or bilateral, we contacted the corresponding author of the paper to clarify. If no clarification was obtained, this field was indicated as “Unknown”. In some cases assumptions, interpretations and slight modifications were made to give data similar formats, and we followed specific rules to ensure consistency throughout the data entry process. Details about this can be found in Supplementary File 6.

### Sharing the database through the EBRAINS Knowledge Graph

We exported .csv files from all the tables in the database. In addition, we made and exported query tables containing selected metadata elements from multiple tables for quantitative estimates, distributions, and cell morphologies. We also created a version of the database specifically designed to input data, and an Excel sheet configured for converting data from any density unit to volumetric densities or bilateral counts to unilateral ones. All of these elements (.csv files, empty database version, and Excel conversion sheet) are shared under a single dataset through EBRAINS^25^.

## Data Records

**Fig. 4:**
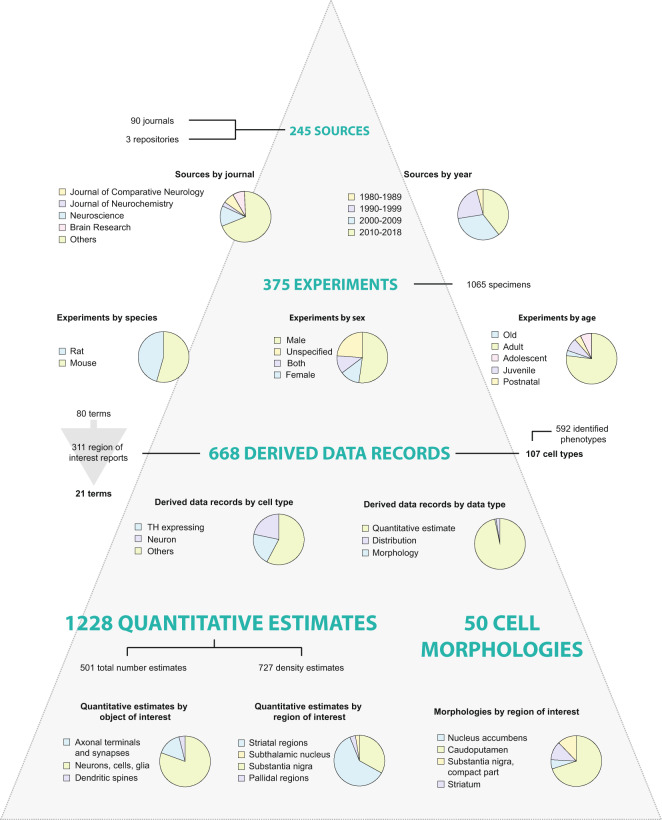
Summary of database content. The current version of the database contains 1228 quantitative estimates and 50 cell morphologies, obtained from 375 experiments reported in 245 sources. Contents are sorted and displayed in pie charts according to key metadata elements. The most common journal sources include the Journal of Comparative Neurology, Journal of Neurochemistry, Neuroscience, and Brain Research. Most sources were published in 2000 or later. Experiments reported are primarily from male, adult animals, with slightly more mouse data included compared to rat data. Through our translation process for anatomical location metadata, 80 anatomical terms were translated to 21 terms found in standard rat and mouse atlases. Data on a total of 100 cell types are included in the database, with approximately half of the 668 derived data records related to TH positive neurons or all neurons. Of the 1228 quantitative estimates found in the database, 501 are total number estimates and 727 are density estimates. These are mainly estimated numbers of neurons, cells or glia, with substantia nigra and striatal regions heavily represented. The 50 cell morphologies in the database are mainly from striatal regions (nucleus accumbens, caudoputamen, or striatum overall). Additional metadata are represented in the database, which is available from the EBRAINS Knowledge Graph^25^.

The database created here, hereafter referred to as the “Murine basal ganglia database”, is shared via EBRAINS^25^ (https://ebrains.eu). It contains information from 375 experiments reported in 245 sources; from these, we extracted 1228 quantitative estimates (501 total number estimates and 727 density estimates), 50 neuronal morphologies, and 18 distribution records of basal ganglia cellular parameters. The content of the Murine basal ganglia database is summarised in Fig. 4.

The shared dataset includes .csv files for all tables in the Murine basal ganglia database as well as the original.accdb file; these files contain the full set of metadata collected during the creation of the database. In addition, we share .csv and .xlsx files for data extracted from the database. These files contain all the numerical, distribution and morphology data available from the Murine basal ganglia database, with selected metadata that we considered relevant for most users. Furthermore, we have established a workflow to allow other researchers to expand upon the knowledge contained in the current version (detailed in usage notes below). To support the use of this workflow, we share an empty version of the Murine basal ganglia database (.accdb) with a spreadsheet (.xlsx), through which researchers can collect and / or contribute more information.

## Technical Validation

In the following, we first consider how the search strings and selection criteria have affected the results of the PubMed search and content of the database. We then evaluate the validity of the graph data extraction procedure. Lastly, we assess and discuss the variability of a selection of the data contained in our database by summarising the information available about the number of tyrosine hydroxylase (TH) positive neurons in the substantia nigra, and the total number of neurons in the caudoputamen.

### Selected papers and repositories

The most common reason for excluding papers were that they did not contain data of interest (54% of papers excluded) or that data were from experimentally manipulated animals without inclusion of a normal control group (15% of papers excluded). Among the studies in which relevant quantitative data had been obtained, 40–45% were excluded in each iteration of the search (11% of all papers) because data were not possible to normalise, due to lack of metadata necessary for comparing the data across studies or re-using them in a different context. Examples include papers where numbers were expressed per section or as percentage of control, or in rare cases, without specification of the unit of measurement. 8% of all search results were excluded because data were presented in graphs only, in each search this concerned 48–59% of the papers of interest with data that otherwise could have been normalised to a common unit of measurement. In a limited selection of papers presenting some data in text and other data in graphs we converted graph data to numeric data (see, Methods) to increase the amount of data extracted, but as this was time consuming it was not feasible to perform on a larger collection of data. In the end, 6% of papers were included. The percentages described here are based on data from the second and third search; the proportions of papers excluded based on the various criteria were relatively similar for the first search, except that the included percentage (3%) was lower since only completely untreated adult animals were included.

Searching and screening papers manually is a time consuming task, and in our literature search led to exclusion of more than 90% of papers. We observe that other literature mining projects have presented similar exclusion rates^67^. This illustrates that designing search strings that are both sensitive and specific is a significant challenge.

### Validation of data extracted from graphs

Papers from the first iteration of the search that presented the same numbers both in graphs and text were used to validate the graph extraction approach. For these cases, we extracted the numbers and error measurements using the graph plot digitiser (see Methods), and compared the resulting numbers with those presented in the text. This showed a negligible discrepancy between means extracted from text and graph (0.08–1% difference), and relatively low differences between extracted standard errors (5–12% difference).

### Variability in a selection of quantitative estimates from the murine basal ganglia

We here present summary data from some of the parameters available in the Murine basal ganglia database. To assess whether variance could be considerably reduced by selecting data obtained by certain methods, we sequentially filtered the data according to methodological metadata (see sections below for details).

#### Tyrosine hydroxylase positive neurons in the substantia nigra

The principal neurons of the substantia nigra are dopaminergic neurons, which can be visualised by using antibodies against the enzyme tyrosine hydroxylase. TH neurons contribute to motor behaviour by their projections to the striatum, and are frequently investigated in murine models for mechanisms of Parkinson’s disease^26^.

**Fig. 5:**
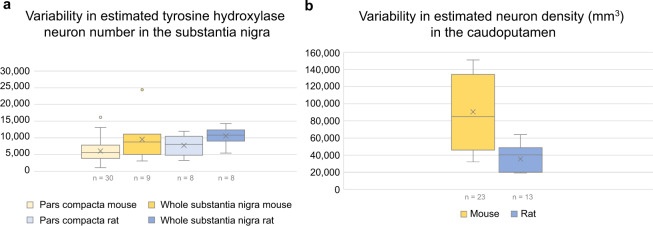
Variability of estimates from the literature. Figure showing summary data for estimates from the database. The whiskers represents the values that fall within 1.5 times the interquartile range. (**a**) Box plot showing total number estimates of TH positive cells in one hemisphere of the substantia nigra pars compacta (light yellow and light blue boxes for mouse and rat, respectively) and the whole substantia nigra (including the compact, reticular and lateral parts, which in some studies also may have included parts of the ventral tegmental area; dark yellow and dark blue boxes for mouse and rat, respectively). (**b**) Box plot showing neuron density estimates in the caudoputamen of mice (yellow box) and rat (blue box). The *n* represents the number of estimates, but note that more than one of these could originate from the same publication or repository source.

In our database, unilateral estimates of the total number of TH neurons in the substantia nigra, pars compacta of the adult (P56 and older) C57BL/6 mouse range from 1090 to 16145 (mean = 6065, SD = 3456, n = 30 estimates). The same range and very similar variation is seen when selecting only stereological studies (range = 1090 to 16145, mean = 6495, SD = 3503, n = 26 estimates). Further filtering of stereological estimates by excluding those that are anatomically non-specific or only partly covering the pars compacta, does not reduce variation either (range = 3360 to 16145, mean = 7706, SD = 3680, n = 14 estimates). Only two of the 30 total number estimates for the C57BL/6 mouse substantia nigra, pars compacta are connected to an antibody with a unique RRID; filtering results based on the exact primary antibody used is therefore not possible. For the adult (P60 and older) rat (all strains), the range of unilateral values in the database is 3260 to 11969 (mean = 7733, SD = 3252, n = 8 estimates). Box plots summarizing these estimates and similar ones from the whole substantia nigra are given in Fig. 5a.

#### Neuron numbers and densities in the caudoputamen

The caudate-putamen complex (hereafter referred to as the caudoputamen) is the largest part of the basal ganglia, receiving axonal projections from the cerebral cortex, and extending projections to several other parts of the basal ganglia circuitry^68^. There are two main types of principal neurons in the caudoputamen, identifiable by the different types of dopamine receptors they possess^26^. Because well-validated and replicated antibodies against these receptors are lacking^69^, studies of the caudoputamen frequently assess total neuron numbers using histochemistry or neuronal markers such as NeuN antibodies.

Unilateral estimates in the database representing the total number of neurons in the caudoputamen of adult mice (all strains) range from 856649 to 1711615 (mean = 1107325, SD = 296707, n = 10 estimates). Note that six of these estimates come from the same study. Estimates of neuron density range from 32166 to 151112 per cubic millimetre (mean = 90407, SD = 42133, n = 23 estimates). The range of density estimates is the same and variability not reduced by selecting stereological estimates only (mean = 88705, SD = 44009, n = 18 studies). In rats, only very few estimates of total numbers for the caudoputamen are available in the database. The estimated neuron density in the adult rat caudoputamen varies from 19129 to 64050 neurons per cubic millimetre (mean = 35529, SD = 15029, n = 13 estimates). The neuron density estimates for caudoputamen are summarised in box plots in Fig. 5b.

#### Possible reasons for observed variability

Our assessment of the variability of quantitative neuroanatomical data from the substantia nigra show that for TH expressing neurons in the pars compacta of C57BL/6 mice, the reported numbers range from approximately 1000 to over 16,000 cells unilaterally. High variability is also seen in the caudoputamen data. Interestingly, the reported neuron density (per cubic millimetre) is on average ~twice as high in the mouse than in the rat. Although the numbers reported within the species varies a lot, the ratio between the mean densities correspond well with estimated scaling rules between rat and mouse brains^70^. For the mouse caudoputamen, estimates of total neuron numbers in the database range from 856649 to 1711615 in one hemisphere. Few studies include the range of values collected in addition to summary statistics, but it is clear that the variability between studies is much higher than that within studies. For example, in a study comparing neuron numbers in the caudoputamen across different mouse strains^71^, the difference between the bilateral average of the groups with the highest and lowest number was 324926. It is thus highly unlikely that the range we observe across studies, of almost one million cells unilaterally, can be attributed solely to biological variance. Instead, the reasons for the large variation in numbers reported from within a region are likely to be manifold. Due to the wide-spread lack of methodological metadata in papers, the size of groups containing estimates obtained by clearly defined and similar methods was too small to support formal statistical analysis on differences in variability. Collection of more data to the Murine basal ganglia database, combined with improved reporting practices, could allow such analyses in the future. Nevertheless, we believe that the present data collection, shared through the EBRAINS Knowledge Graph, can be useful for finding and comparing published data. The ability to filter the data based on metadata elements might also be useful to select appropriate data, depending on the need of the user. Combined with our defined workflows for contributing more information, we believe these results will make it easier to select, organise, compare and share quantitative information from the literature or from new analyses in the future. We describe these uses of the database in detail below.

## Usage Notes

Our database is shared through the EBRAINS Knowledge Graph as part of a dataset entitled “Database of quantitative cellular and subcellular morphological properties from rat and mouse basal ganglia”^25^. It comprises three main parts (see Data records for details): 1) the Murine basal ganglia database (Database_v1.accdb); 2) spreadsheets with all the quantitative estimates, morphologies and distributions contained in the Murine basal ganglia database (files in .xlsx and .csv format) with selected metadata; and 3) an empty version of the Murine basal ganglia database (Input_database.accdb) and a spreadsheet (Input_sheet .xlsx) facilitating collection of new data. We here briefly explain how researchers with different interests may utilise different parts of this dataset. These descriptions are intended as example use cases, and the reader is referred to other sources^72,73^ for guides on the use of Microsoft Access and Excel (RRID:SCR_016137). Because maintaining and updating information is a challenge with any database that is seldom addressed, we go on to describe a workflow through which other researchers can organise and share more data, using shared database and spreadsheet templates.

### Using the Murine basal ganglia database and the data extracted from it

#### Exploring the reported numbers of tyrosine hydroxylase neurons in substantia nigra

A researcher wants to look up the reported numbers of TH neurons in substantia nigra. Having downloaded the dataset titled “Database of quantitative cellular and subcellular morphological properties from rat and mouse basal ganglia”^25^, (s)he opens the README file to get a quick overview of the contents. There, (s)he sees that the Data Extracts-folder contains queries that include all the numbers available in the database. (S)he finds that such extracts are likely to meet his/her questions, and navigates to the relevant folder. S(h)e opens the.xlxs file called “Cell counts” and selects the sheet called “Total number estimates”. The first three columns show the cell types that have been quantified, the species, and the regions of interest. The researcher filters the records to “Tyrosine hydroxylase expressing” cells, “Mus musculus” and “Pars compacta” (to simultaneously filter multiple columns in Microsoft Excel, select all the columns to be filtered, and under the Data tab click “Filter”). This yields 70 records, each one with accompanying metadata elements related to the animals, counting method, and region of interest. To explore the data further, e.g. by extracting descriptive measurements, (s)he copies the filtered records to a new sheet (in Microsoft Excel, go to Find & Select, click “Go to special” and select “Visible cells only”).

#### Finding studies using a specific primary antibody

A researcher has used immunohistochemistry to visualize parvalbumin positive neurons, and quantified labelled cells using stereological analysis. To verify the results (s)he is now interested in finding quantitative data from studies where the same antibody has been used. (S)he downloads the dataset titled “Database of quantitative cellular and subcellular morphological properties from rat and mouse basal ganglia”^25^ from EBRAINS, and upon looking at the “Cell counts” data extracts finds that they do not contain metadata specifying the antibody used. (S)he therefore navigates to the Database-folder and opens the.accdb file and the text file called “Tables_description”. In the text file, (s)he finds that antibodies are stored in the lookup table “Reporters” with connections to the table “Sources” via other tables. (S)he navigates to the Create table and clicks the Query Wizard. After selecting the Simple Query Wizard, (s)he selects the “Reporter name” and “Reporter unique ID” fields from the “Reporters” table and the “Source name” and “Source ID” field from the “Sources” table. (S)he clicks “Finish” and is presented with a query including a list of antibodies, their unique RRIDs, and the name and DOI of the source in which they were used. (S)he clicks the “Reporter unique ID” column header and scrolls to see if the antibody of interest (RRID:AB_10000344) is among the listed IDs. It is, and (s)he filters the list to these records. This yields a list of four studies where the antibody has been used. The researcher can now look for results from these studies in the Cell counts data extract by filtering it to the relevant Source names, or look up the original papers by use of the DOIs.

#### Overviewing the methodological parameters of a study

Upon identifying the studies using an antibody of interest, the researcher described above wants to get a quick overview of the methods used in these studies. In the Tables description-file, (s)he reads that the tables “Specimens” and “Specimen_treatments” contain information related to the treatment of tissue reported in included papers. (S)he therefore creates a new query, including the “Source name” from the “Sources” table, the “Specimen form” from the “Specimens” table and the fields “Solution”, “Purpose”, “Time”, “Time unit” and “Temperature” from the “Specimen treatments” table. Since (s)he is primarily interested in the methods related to parvalbumin stained material, (s)he also includes the “Cell type putative” field from the table “Derived data records”. She clicks “Finish”, and filters the resulting query to his/her studies of interest by clicking the “Source name” column header and selecting the relevant Source names. (S)he also clicks the “Cell type putative” column header and uses the text filter to select only records that contains “Parvalbumin”. This yields 13 records summarizing the treatments used for each specimen.

### Using the workflow for harvesting, organizing and updating neuroanatomical data

**Fig. 6:**
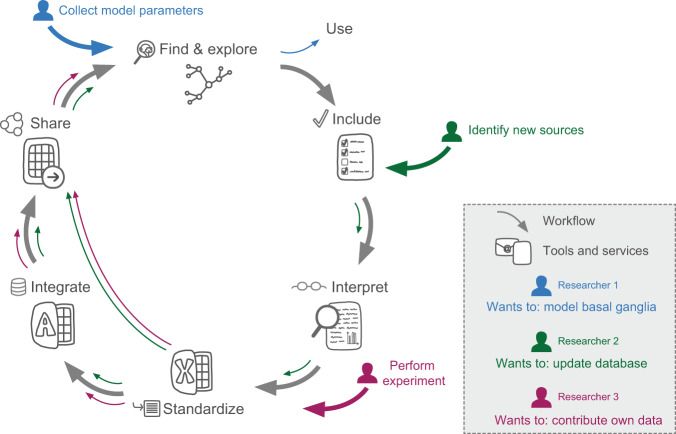
Workflow for integration of derived data. Schematic representation of the main workflow described in this paper, as well as the tools and services used (in grey). Coloured arrows indicate specific ways different users may interact with the database or the workflow, today or in the future. Blue arrows illustrate how a researcher may use the database to identify data to constrain a computational model of the basal ganglia. Through the EBRAINS Knowledge Graph, derived data may be found as a shared dataset^25^, downloaded and explored. Green and purple arrows show how researchers may use the workflow and resources developed to update the database in the future, identifying and interpreting new sources (green arrows) or by contributing own data (purple arrows). In the workflow, data are included by use of specific criteria, and interpreted manually. Derived data are extracted and standardised to common units of measurement using a custom Excel workbook. The Excel workbook is designed for input of data as well as minimum metadata according to the EBRAINS standard. Optionally, more extensive metadata may be integrated through a Microsoft Access database input portal. Once organised, derived data may be shared through the EBRAINS curation support, through which it can be made available as part of the collection found in the Knowledge Graph.

In order to compare numbers reported across studies, it is first necessary to systematically extract relevant data and metadata and to standardise these to common units and concepts. We here present a workflow enabling users to harvest and organise their quantitative neuroanatomical data from the literature or public databases. This workflow includes a template version of the Murine basal ganglia database (with forms supporting input of largely standardised metadata) available through the dataset hosted in the EBRAINS Knowledge Graph^25^, a novel procedure for translating terms for regions of interest to common terminology, and a preliminary approach for mapping data to cell-types of interest. We include steps through which other researchers can enter new information to the database and share this with the broader community. The workflow is summarised in Fig. 6.

#### Translation of terms across neuroanatomical nomenclatures

A key part of the overall workflow is the translation of semantic terms existing in different reference atlases to terms used in the standard reference atlases used by EBRAINS (Supplementary Fig. 1). These include the Waxholm Space atlas of the Rat Brain^34,35,74^ and the Allen Mouse Brain Common Coordinate Framework^37^. Anatomical metadata found in sources essentially enters one of three routes. In the first route, terms that are consistent with the nomenclature for one of the EBRAINS standard atlases are directly entered in the database. In the second route, terms that are consistent with another atlas nomenclature are translated to the closest matching region term in the relevant EBRAINS atlas. The basis for making such a translation is given by the spatial relationships between regions delineated in the different atlases used, and regions in the EBRAINS standard atlases (see Methods for details); these relationships are available as datasets through the EBRAINS Knowledge Graph^61,62^. The third route for inserting anatomical metadata found in sources, are for terms that are not consistent with any standard nomenclature. These are treated as “custom” terms, and translated to the closest EBRAINS atlas term by using the documentation available from the source. This was the most commonly used route in the current project, used for ~62% of reported regions. Note that 4% of the sources used an atlas for which relationships to EBRAINS atlases were not available, and these also entered the custom region translation route.

#### Updating the Murine basal ganglia database

A database is only up-to-date as long as the data are maintained and expanded with new information. In addition to sharing the content aggregated and organised through this project, we therefore outline how researchers could contribute to the Murine basal ganglia database in the future. Researchers might want to add more data from the literature (green arrows, Fig. 6) or from own experiments (purple arrows, Fig. 6). The first step for anyone wishing to add more data from the published literature is to identify potential new sources. This could be done through a literature search similar to that described in this paper (with date filters constraining the search to the period after the current search was performed, see Supplementary File 2 for a list of the search strings used here). Alternatively, advances in text mining might yield opportunities for more automatic search strategies^75^. The next step in the workflow is interpretation: the source needs to be examined manually to identify the relevant data and metadata elements to be extracted. Once produced or identified through the literature, data should be extracted and metadata standardised. For this purpose, we share an Excel workbook with sheets where data can be entered in any format. Upon insertion of the number and unit, calculated fields standardise data to represent number per square or cubic micro- or millimetre. For cell counts, volumetric densities are also estimated from 2D counts given that section thickness is provided, according to the calculations described in Supplementary File 5. The Excel workbook is also tailored for input of basic metadata as required by EBRAINS (https://ebrains.eu/). It may be used in a relatively simple route to collecting data and contributing to the database (long green and purple lines in Fig. 6). Alternatively, it may be used as a means to organise and standardise data before entering it with extended metadata in the database. For this purpose, we share an empty version of the Murine basal ganglia database (an “input portal”) specifically designed to add new data with the full extent of metadata collected for the current project. The Excel sheet and Access database tailored for input are shared as .xlxs and .accdb files, respectively, and can be downloaded together with the Murine basal ganglia database through the dataset hosted at EBRAINS Knowledge Graph^25^. In our experience, the interpretation, extraction, standardisation and integration steps might require from half an hour to several hours per publication; generally, less time is required to integrate data that is provided in tabular format and using standard units of measurement (square or cubic micro- or millimetres), with a clearly described methodology. The files shared here to facilitate collection of new data may be populated and stored locally by the user, or shared with the community. The last step of the workflow outlined here (Fig. 6) is thus sharing the data. This could be done through any data sharing platform, e.g. Zenodo (www.zenodo.org) or Figshare (www.figshare.com). The EBRAINS curation service (curation-support@ebrains.eu) and Knowledge Graph offers the advantage of being tailored to neuroscience data, and would allow for new data collections to be linked to the version of the Murine basal ganglia database presented here^25^.

## Supplementary information

Supplementary file 1

Supplementary file 2

Supplementary file 3

Supplementary file 4

Supplementary file 5

Supplementary file 6

Supplementary figure 1

## Data Availability

The QuickNII (RRID:SCR_016854) tool was used for spatial co-registration of atlases. Microscoft Access 2016 was used to create the database.

## References

[1] Egger R, Dercksen V, Udvary D, Hege H-C, Oberlaender M (2014). Generation of dense statistical connectomes from sparse morphological data. Front. Neuroanat..

[2] Markram H (2015). Reconstruction and simulation of neocortical microcircuitry. Cell.

[3] Bezaire MJ, Soltesz I (2013). Quantitative assessment of CA1 local circuits: knowledge base for interneuron-pyramidal cell connectivity. Hippocampus.

[4] Abercrombie M (1946). Estimation of nuclear population from microtome sections. Anat. Rec..

[5] Schmitz C, Hof P (2005). Design-based stereology in neuroscience. Neuroscience.

[6] Brændgaard H, Gundersen HJG (1986). The impact of recent stereological advances on quantitative studies of the nervous system. J. Neurosci. Methods.

[7] Bjaalie J, Diggle P, Nikundiwe A, Karagulle T, Brodal P (1991). Spatial segregation between populations of ponto-cerebellar neurons: Statistical analysis of multivariate spatial interactions. Anat. Rec..

[8] Prodanov D, Nagelkerke N, Marani E (2007). Spatial clustering analysis in neuroanatomy: Applications of different approaches to motor nerve fiber distribution. J. Neurosci. Methods.

[9] West MJ, Østergaard K, Andreassen OA, Finsen B (1996). Estimation of the number of somatostatin neurons in the striatum: An *in situ* hybridization study using the optical fractionator method. J. Comp. Neurol..

[10] Oorschot D (1996). Total number of neurons in the neostriatal, pallidal, subthalamic, and substantia nigral nuclei of the rat basal ganglia: A stereological study using the cavalieri and optical disector methods. J. Comp. Neurol..

[11] Yu, Z. *et al*. Nitrated α-synuclein induces the loss of dopaminergic neurons in the substantia nigra of rats. *PLoS One* **5**, (2010). 10.1371/journal.pone.0009956 PMC2851648 20386702

[12] Singh A (2012). Long term exposure to cypermethrin induces nigrostriatal dopaminergic neurodegeneration in adult rats: postnatal exposure enhances the susceptibility during adulthood. Neurobiol. Aging.

[13] Bornmann L, Mutz R (2015). Growth rates of modern science: A bibliometric analysis based on the number of publications and cited references. J. Assoc. Inf. Sci. Technol..

[14] van Strien NM, Cappaert NL, Witter MP (2009). The anatomy of memory: an interactive overview of the parahippocampal-hippocampal network. Nat Rev Neurosci.

[15] Bota M, Dong H-W, Swanson L (2005). Brain Architecture Management System. Neuroinformatics.

[16] Wheeler D (2015). Hippocampome.org: a knowledge base of neuron types in the rodent hippocampus. Elife.

[17] Ascoli G, Donohue D, Halavi M (2007). NeuroMorpho.Org: A central resource for neuronal morphologies. J. Neurosci..

[18] Erö C, Gewaltig M, Keller D, Markram H (2018). A cell atlas for the mouse brain. Front. Neuroinform..

[19] Kim Y (2017). Brain-wide maps reveal stereotyped cell-type-based cortical architecture and subcortical sexual dimorphism. Cell.

[20] Murakami T (2018). A three-dimensional single-cell-resolution whole-brain atlas using CUBIC-X expansion microscopy and tissue clearing. Nat. Neurosci..

[21] Obeso J (2009). The basal ganglia in Parkinson’s disease: Current concepts and unexplained observations. Ann. Neurol..

[22] Bunner KD, Rebec GV (2016). Corticostriatal dysfunction in Huntington’s disease: The basics. Front. Hum. Neurosci..

[23] Vidyadhara DJ, Yarreiphang H, Raju TR, Alladi PA (2017). Admixing of MPTP-resistant and susceptible mice strains augments nigrostriatal neuronal correlates to resist MPTP-induced neurodegeneration. Mol. Neurobiol..

[24] Baquet Z, Williams D, Brody J, Smeyne R (2009). A comparison of model-based (2D) and design-based (3D) stereological methods for estimating cell number in the substantia nigra pars compacta (SNpc) of the C57BL/6J mouse. Neuroscience.

[25] Bjerke I, Puchades M, Bjaalie JG, Leergaard T (2019). Human Brain Project Neuroinformatics Platform.

[26] Gerfen CR, Bolam JP (2016). The neuroanatomical organization of the basal ganglia. Handb. Behav. Neurosci..

[27] Olmos J, Heimer L (1999). The concepts of the ventral striatopallidal system and extended amygdala. Ann. N. Y. Acad. Sci..

[28] Gupta A (2008). Federated access to heterogeneous information resources in the neuroscience information framework (NIF). Neuroinformatics.

[29] Polavaram S, Gillette T, Parekh R, Ascoli G (2014). Statistical analysis and data mining of digital reconstructions of dendritic morphologies. Front. Neuroanat..

[30] Yates S, Puchades M (2019). Human Brain Project Neuroinformatics Platform.

[31] Martone M (2008). The Cell Centered Database project: An update on building community resources for managing and sharing 3D imaging data. J. Struct. Biol..

[32] Paxinos, G. & Watson, C. *The rat brain in stereotaxic coordinates*. (Elsevier Inc (2013). 10.1016/0165-0270(80)90021-7 6110810

[33] Swanson, L. *Brain Maps III: Structure of the rat brain*. (Elsevier (2004).

[34] Papp E, Leergaard TB, Calabrese E, Johnson GA, Bjaalie JG (2014). Waxholm Space atlas of the Sprague Dawley rat brain. Neuroimage.

[35] Kjonigsen L, Lillehaug S, Bjaalie J, Witter M, Leergaard T (2015). Waxholm Space atlas of the rat brain hippocampal region: Three-dimensional delineations based on magnetic resonance and diffusion tensor imaging. Neuroimage.

[36] Paxinos, G. & Franklin, K. *The mouse brain in stereotaxic coordinates*. (Academic Press (2012).

[37] Oh S (2014). A mesoscale connectome of the mouse brain. Nature.

[38] Papp EA, Leergaard TB, Calabrese E, Allan Johnson G, Bjaalie JG (2015). Addendum to “Waxholm Space atlas of the Sprague Dawley rat brain” [NeuroImage 97 (2014) 374-386]. Neuroimage.

[39] Puchades, M., Csucs, G., Ledergerber, D., Leergaard, T. & Bjaalie, J. Spatial registration of serial microscopic brain images to three-dimensional reference atlases with the QuickNII tool. *PLoS One* **14**, (2019). 10.1371/journal.pone.0216796 PMC6541252 31141518

[40] Franklin, K. & Paxinos, G. *The mouse brain in stereotaxic coordinates*. (Academic Press (1996).

[41] Franklin, K. & Paxinos, G. *The mouse brain in stereotaxic coordinates*. (Academic Press (2007).

[42] Paxinos, G. & Watson, C. *The rat brain in stereotaxic coordinates*. (Academic Press (1998).

[43] Paxinos, G. & Watson, C. *The rat brain in stereotaxic coordinates*. (Academic Press (2007).

[44] Paxinos, G. & Watson, C. *The rat brain in stereotaxic coordinates*. (Academic Press (1986).

[45] Paxinos, G. & Watson, C. *The rat brain in stereotaxic coordinates*. (Elsevier (2005). 10.1016/0165-0270(80)90021-7 6110810

[46] Swanson, L. *Brain Maps: Structure of the rat brain*. (Elsevier (1992).

[47] Swanson, L. *Brain Maps II: Structure of the rat brain*. (Elsevier (1998).

[48] Bjerke I, Schlegel U, Puchades M, Bjaalie J, Leergaard T (2019). Human Brain Project Neuroinformatics Platform.

[49] Bjerke I, Schlegel U, Puchades M, Bjaalie J, Leergaard T (2019). Human Brain Project Neuroinformatics Platform.

[50] Bjerke I, Schlegel U, Puchades M, Bjaalie J, Leergaard T (2019). Human Brain Project Neuroinformatics Platform.

[51] Bjerke I, Schlegel U, Puchades M, Bjaalie J, Leergaard T (2019). Human Brain Project Neuroinformatics Platform.

[52] Bjerke I, Schlegel U, Puchades M, Bjaalie J, Leergaard T (2019). Human Brain Project Neuroinformatics Platform.

[53] Bjerke I, Schlegel U, Puchades M, Bjaalie J, Leergaard T (2019). Human Brain Project Neuroinformatics Platform.

[54] Bjerke I, Schlegel U, Puchades M, Bjaalie J, Leergaard T (2019). Human Brain Project Neuroinformatics Platform.

[55] Bjerke I, Schlegel U, Puchades M, Bjaalie J, Leergaard T (2019). Human Brain Project Neuroinformatics Platform.

[56] Bjerke I, Schlegel U, Puchades M, Bjaalie J, Leergaard T (2019). Human Brain Project Neuroinformatics Platform.

[57] Bjerke I, Schlegel U, Puchades M, Bjaalie J, Leergaard T (2019). Human Brain Project Neuroinformatics Platform.

[58] Bjerke I, Schlegel U, Puchades M, Bjaalie J, Leergaard T (2019). Human Brain Project Neuroinformatics Platform.

[59] Bjerke I, Schlegel U, Puchades M, Bjaalie J, Leergaard T (2019). Human Brain Project Neuroinformatics Platform.

[60] Bjerke I, Schlegel U, Puchades M, Bjaalie J, Leergaard T (2019). Human Brain Project Neuroinformatics Platform.

[61] Bjerke I, Puchades M, Bjaalie J, Leergaard T (2019). Human Brain Project Neuroinformatics Platform.

[62] Bjerke I, Puchades M, Bjaalie J, Leergaard T (2019). Human Brain Project Neuroinformatics Platform.

[63] Hamilton D (2017). Name-calling in the hippocampus (and beyond): coming to terms with neuron types and properties. Brain Informatics.

[64] Ascoli G (2008). Petilla terminology: nomenclature of features of GABAergic interneurons of the cerebral cortex. Nat. Rev. Neurosci..

[65] Keller D, Erö C, Markram H (2018). Cell densities in the mouse brain: A systematic review. Front. Neuroanat..

[66] Coggeshall R (1992). A consideration of neural counting methods. Trends Neurosci..

[67] Sugar J, Witter M, van Strien N, Cappaert N (2011). The retrosplenial cortex: intrinsic connectivity and connections with the (para)hippocampal region in the rat. An interactive connectome. Front. Neuroinform..

[68] Voorn P, Vanderschuren L, Groenewegen H, Robbins T, Pennartz C (2004). Putting a spin on the dorsal-ventral divide of the striatum. Trends Neurosci..

[69] Cullity, E., Madsen, H., Perry, C. & Kim, J. Postnatal developmental trajectory of dopamine receptor 1 and 2 expression in cortical and striatal brain regions. *J. Comp. Neurol*. 1–17 (2018). 10.1002/cne.24574 30408161

[70] Herculano-Houzel S, Mota B, Lent R (2006). Cellular scaling rules for rodent brains. Proc. Natl. Acad. Sci. U. S. A..

[71] Rosen GD, Williams RW (2001). Complex trait analysis of the mouse striatum: independent QTLs modulate volume and neuron number. BMC Neurosci..

[72] Barrows, A., Young, M. & Stockman, J. *Microsoft Access 2010 all-in-one for dummies*. (Wiley Publishing (2010).

[73] Frye, C. *Microsoft Excel 2019*. (Microsoft Press (2019).

[74] Osen K, Imad P, Wennberg A, Papp E, Leergaard T (2019). Waxholm Space atlas of the rat brain auditory system: Three-dimensional delineations based on structural and diffusion tensor magnetic resonance imaging. Neuroimage.

[75] French L (2015). Text mining for neuroanatomy using WhiteText with an updated corpus and a new web application. Front. Neuroinform..

