# Supplementary material for: Database of literature derived cellular measurements from the murine basal ganglia
Source: Sci Data. 2020 Jul 6;7:211. doi: 10.1038/s41597-020-0550-3 (PMC7338524; doi:10.1038/s41597-020-0550-3)
Supplement: Supplementary file 1 [file 41597_2020_550_MOESM1_ESM.pdf]

Tables in the database

Table of Contents

1. Sources....3
2. Experiments....3
3. Specimen related tables....4
   3.1. Specimens....4
   3.2. Sectioning details....4
   3.3. Specimen treatments....5
   3.4. Reporter incubations....5
   3.5. Single cell labelings....5
4. Derived data record related tables....6
   4.1. Derived data records....6
   4.2. Cell type classifications....7
   4.3. Electron microscopy details....7
   4.4. Light / fluorescence microscopy details....7
   4.5. Cell morphologies....8
   4.6. Quantitations....8
   4.7. Distributions....9
   4.8. Stereology details....10
5. Region records and related tables....11
   5.1. Region records....11
   5.2. Original region records....12
6. Other tables....12
   6.1. Calculations....12
   6.2. Considered papers....13
   6.3. Considered papers decisions....13
   6.4. Lookup tables....13
      6.4.1. Age categories....13
      6.4.2. Cell phenotype categories....14
      6.4.3. Cell phenotypes....14
      6.4.4. Cellular regions....14
      6.4.5. Cell types....14

6.4.6. Microscopes 14
6.4.7. Nomenclatures other 14
6.4.8. Nomenclatures preferred 15
6.4.9. Objects of interest 15
6.4.10. Regions 15
6.4.11. Regions other 15
6.4.12. Region zones 15
6.4.13. Reporters 16
6.4.14. Reporter labels 16
6.4.15. Reporter targets 16
6.4.16. Sectioning instruments 16
6.4.17. Sex 16
6.4.18. Softwares 16
6.4.19. Solutions 17
6.4.20. Source origins lookup 17
6.4.21. Species 17
6.4.22. Strains 17
6.4.23. Substrains 17
6.4.24. Transgenic lines 17
6.4.25. Visualization methods 17
6.5. Connecting tables 18
6.5.1. Derived data_EMdetails 18
6.5.2. Derived data_LFMdetails 18

Note: All tables have automatically incrementing numbers as primary keys unless otherwise specified.

1. Sources

The sources table contains information about the source of derived data entered in the database. Derived data can come from either a publication or a dataset in a repository. The table has the following fields:

ID (primary key): The unique identifier of the source, either DOI, a PMID or an RRID in the case of a database source.

Source name: The name given to the source in the database. For a publication, this is formatted as [name of first author]_[year]. For a repository, the format is [name of repository]_[name of dataset].

Inserted date: The date of entry in the database

Source type: The type of source, either “Publication” or “Database”

Source publication year: Year of publication or database

Source origin: Name of either the journal (for publications) or the repository (for repositories), indexes ID in the table “Source origins lookup”

Source title: The title of the publication or dataset

Raw data available: Whether or not raw data are available

2. Experiments

A source may have one or more experiments, information about which is stored in the experiments table. It has the following fields:

Experiment name: The name given to the experiment, formatted as [source name]. If more than one experiment is available from a single experiment, a descriptive string is added to separate experimental groups.

Source: Foreign key, referencing ID in sources table

Species: Species of the animals, indexing the lookup table “Species”

Strain: Strain of the animals, e.g. C57BL/6, indexing the lookup table “Strains”

Substrain: The specific substrain of the animal, e.g. C57BL/6J, indexing the lookup table “Substrains”

Animal status: The status of the animal, according to the type of treatment they have been exposed to.

Transgenic line name: Transgenic line of the animal, indexing the lookup table “Transgenic line”

Sex: Sex of the animals, indexing the lookup table “Sex”

Age lower limit: Lowest age of animals used in the experiment in days

Age upper limit: Highest age of animals used in the experiment in days

Age: The developmental stage of the animal, indexing the lookup table “Age categories”

Weight lower limit: Lowest weight of animals used in the experiment in grams

Weight upper limit: Highest weight of animals used in the experiment in grams

Anesthetic: The anesthetic agent used in the experiment, indexing the lookup table “Solutions”

Perfusion fix medium: The perfusion fixation medium used in the experiment, indexing the lookup table “Solutions”

3. Specimen related tables

3.1. Specimens

An experiment may give rise to one or more specimens. A specimen is in this context defined as a group of tissue samples with similar form and which has undergone similar treatment. For example, a group of brains used in an experiment would constitute one specimen. If tissue sections were cut from these brains, those would together constitute another specimen. These specimen are not linked directly, but would both be linked to the same experiment and annotated as "primary" and "secondary" in another field (“Specimen order”, see below). There could also be "tertiary" and "quarternary" specimen, e.g. pieces dissected from a section or ultrathin sections for electron microscopy. The specimens table has the following fields:

Specimen name: The name given to the specimen, formatted as [experiment name]_[specimen form]. In the case of several specimens of the same form that are treated differently, they have a descriptive string attached in order to be separated.

Experiment: Foreign key, referencing ID in experiments table

Specimen form: The form of the specimen, defined as whole brain, hemisphere, block, slice (>120 µm), mounted thick section, free floating thick section, block from section, semithin section (0.1-1 µm), or ultrathin section (<100 nm).

Specimen order: The order of the specimen.

A specimen may or may not have records in the following tables: sectioning details, specimen treatments, and single-cell labeling details.

3.2. Sectioning details

Specimen: Foreign key, referencing ID in specimens table. Sectioning details are related to the specimens that *have been* cut and not to the specimen it has been cut from.

Section thickness: Thickness of the sections in µm

Sectioning instrument: Type of instrument on which sections have been cut, indexes lookup table "Sectioning instruments"

Section orientation: Orientation of sections, defined as coronal, horizontal, sagittal, or non-standard

3.3. Specimen treatments

Specimen: Foreign key, referencing ID in specimens table.

Solution: The solution in which the specimen has been treated, indexes ID in the lookup table "Solutions".

Purpose: The purpose of the treatment.

Time: The duration of the treatment. "Overnight" is stored as 12 hours, a "day" is considered 24 hours.

Time unit: The unit of duration, defined as minutes, hours, days, or weeks.

Temperature: The temperature in which the treatment was performed.

3.4. Reporter incubations

Incubations in solutions containing antibodies or nucleic acid probes is a specialized form of treatment, and information about these incubations are stored in a separate table from other specimen treatments.

Specimen: Foreign key, referencing ID in specimens table.

Reporter: The reporter used in the incubation. This field indexes the lookup table "Reporters"

Order: The order of the probe, i.e. primary, secondary, etc.

Concentration: The concentration of the probe.

Time: The duration of the incubation in hours. "Overnight" is stored as 12 hours, a "day" is considered 24 hours.

Temperature: The temperature in which the incubation was performed.

3.5. Single cell labelings

Specimen: Foreign key, referencing ID in specimens table.

Visualization medium: The medium injected to visualize the cell, indexes ID in the "Solutions" table.

Medium concentration: Concentration of the injected medium.

Medium volume: The amount of medium injected to visualize the cell.

Introduction timing: Timing of single cell injection, either in vivo or in vitro.

Introduction type: Type of injection, selection of intracellular injection, viral infection, electroporation, intracranial injection, ballistic delivery, or iontoporation.

4. Derived data record related tables

A specimen may have one or more derived data records, information about which is stored in the derived data records table. Importantly, a derived data record can relate to one or more records in one of the following tables: cell morphologies, quantitations, and distributions.

4.1. Derived data records

Derived data record name: The name given to the derived data record, formatted as [specimen name]_[cell type]_[object of interest]_[derived data type]

Specimen: Foreign key, referencing ID in specimens table.

Derived data type: The type of derived data, either cell morphology, quantitation, or distribution

Number of animals: The number of animals used in the analysis that led to the derived dataset

Object of interest: The object of interest. This field indexes the lookup table "Objects of interest"

Cell type putative: The cell type to which the data are thought to belong. More extensive information about the cell phenotype is found in the cell type classifications table, and due to the lack of any universally recognized cell type ontology, this field provides a temporary cell type based on current knowledge and ideas. This field indexes the lookup table "Cell types".

OOI recognition criteria: The criteria used to recognize the object of interest, usually a direct excerpt from the publication.

Visualization method: The method of visualization, indexes the table "visualization methods"

Shrinkage correction: Whether or not shrinkage correction has been performed, a procedure that mainly will affect densities and size measurements.

Cell types can be defined in several ways, and there are not yet any commonly accepted cell type ontologies. A special interest group is currently working on a definition of cell types based on several phenotype categories[1]. This type of classification has been incorporated to the extent possible in the database. Any morphology or quantitation in the database should relate to a cell type; thus, each derived data record is related to one or more records in the cell type classifications table. The information in papers or repositories about the phenotype(s) of cell types are stored here, and can be used later for more proper sorting of data according to cell types.

[1] https://www.incf.org/collaborate/incf-special-interest-groups/incf-sig-on-neuroinformatics-for-cell-types

4.2. Cell type classifications

Derived data record: Foreign key, referencing ID in derived data records table.

Phenotype identified: The phenotype of the cell within the phenotype category. This field indexes the lookup table “Cell phenotypes”, where cell phenotypes are stored with their phenotype category and preferred ontological identifiers found in Interlex.

A derived data record might furthermore have records in either the “Electron microscopy details” table or the “Light / fluorescence microscopy details” table. These are connected to the derived data records table by a many-to-many connection captured in separate tables, because a derived data record might be obtained by use of several microscopes with individual details related to them; conversely, a record in the microscopy tables might be related to several derived data records, because several derived data records might be obtained with the same microscope and similar settings.

4.3. Electron microscopy details

EM details name: Name of EM details, formatted as [derived data record name]_[EMdetails]. If a derived data record has been obtained with several microscopes, a descriptive string should be added to each of the records.

Microscope: The microscope type with which the derived data record was obtained. The field indexes the lookup table “Microscopes”.

Grid type: The grid type, selection of either slot grid or mesh grid

Magnification: The magnification at which the derived data record has been obtained

4.4. Light / fluorescence microscopy details

Microscopy details name: Name of LFM details, formatted as [specimen name]_[LFMdetails].

Microscope: The microscope type with which the derived data record was obtained, indexing the lookup table “Microscopes”.

Mounting medium: The mounting medium that has been used, indexing the lookup table “Solutions”.

Refraction medium: The objective type (refractive medium), selection of air, water or oil

Numerical aperture: The numerical aperture of the objective

Objective lens: The magnification of the objective

Total magnification: The total magnification used

Pixel size: The resolution in the x y plane in µm

Z stack: Whether or not a z stack has been obtained

Optical slice size: The resolution in the z plane in µm

4.5. Cell morphologies

The cell morphologies table mainly contains fields with data derived from NeuroMorpho. Other fields include:

Morphology name: The name of the morphology, formatted as [derived dataset name]_ROI. If several morphologies are related to the same derived data record, their names should be accompanied by a number.

Neuromorpho_ID: The ID of the neuron in the Neuromorpho database

Derived data record: Foreign key, referencing ID in derived data records

Region record: Foreign key, referencing ID in region records

The fields with derived data includes the following for morphological parameters:

Soma surface, Number stems, Number bifurcations, Number branches, Overall width, Overall height, Overall depth, Average branch diameter, Total arbor length, Total arbor surface, Total arbor volume, Max Euclidian distance, Max path distance, Max branch order, Average contraction, Total fragmentation, Partition asymmetry, Average Ralls, Average bifurcation angle local, Average bifurcation angle remote, Fractal dimension

Furthermore, these fields of additional information from NeuroMorpho are included:

Physical integrity: Metadata related to completeness of the reconstruction

Structural domains: Metadata related to completeness of the reconstruction

Morphological attributes: Metadata related to completeness of the reconstruction

Reconstruction method: This field indexes the table "Softwares", thus gives information about the software used for reconstruction

Original format: Original file format of the reconstruction found in NeuroMorpho

4.6. Quantitations

Quantitation name: The name of the quantitation, formatted as [derived data record name]_ROI.

Derived data record: Foreign key, referencing ID in derived data records.

Region zone: The specific zone within the region of interest from which data were taken, e.g. patch or matrix of striatum.

Region record: Foreign key, referencing ID in region records

Cellular target region: Specifies the targeted cellular region, e.g. if synapses made onto dendritic spines have been counted, the cellular target is "dendritic spine". If dendritic spines have been counted on distal dendrites only, the cellular target is "distal dendrite".

Cellular target ID (for axonal terminal and synapse data only): Specifies the cellular ID for the cellular target, referencing the lookup table "Cell types"

Estimate relevance: Specifies whether the number or density is an estimate per cellular origin (e.g. number of synapses made by a cell type specified in putative cell type) or per cellular target (e.g. number of synapses made by a cell type specified in putative cell type *per* a specified cellular target)

Section sampling: The method of sampling sections, defined as exhaustive (all sections), systematic random (random start with uniform fraction), preferential, or N/A.

Sampling fraction: The ratio of sampled to unsampled sections

Subsectional sampling: The method for counting object of interest within a section, defined as "exhaustive, manual", "exhaustive, automatic", "exhaustive, semi-automatic", "subset, automatic", "subset, biased" or "subset, unbiased"

Final estimate basis: The basis of the final estimate

Original extent: Information about whether counts are uni- or bilateral.

Number: The number of cells counted / estimated

Number SD: Standard deviation of the total number estimate

Density: The density of cells counted

Density unit: Unit of density, e.g. mm^2 or mm^3

Density SD: Standard deviation of the density estimate

Software: The software used in the counting process, indexes the lookup table "Softwares"

Stereology details record: Foreign key, referencing ID in the table "Stereology details"

Estimate extraction: Indicates where in the paper the estimate was extracted from, either "From text or table" or "From graph".

4.7. Distributions

Distribution name: The name of the distribution, formatted as [derived data record name]_ROI. If several distributions are related to the same derived data record and region record, their names should be accompanied by a descriptive string.

Derived data record: Foreign key, referencing ID in derived data records.

Region of interest: The region of interest to which the distribution relates

Region record: Foreign key, referencing ID in region records

Section sampling method: The method of sampling sections, defined as exhaustive (all sections), systematic random (random start with uniform fraction), or preferential.

Sampling fraction: The ratio of sampled to unsampled sections

Distribution: Description of the distribution, defined as either "Rostrocaudal increase", "Rostrocaudal decrease", "Mediolateral increase", "Mediolateral decrease", "Dorsoventral increase", "Dorsoventral decrease", "Clustering", "Segregation", or "Random"

Distribution dimensions: The dimensions in which the distribution has been analyzed, either 2D (individual section) or 3D (series of sections with point reconstructions).

Analysis type primary: The overall type of analysis, defined as point based (nearest neighbor, Ripley's K function), count based (variants of quadrant count) or partition based (Voronoi tessellation)

Analysis type secondary: The specific type of analysis, selection of "quadrant count, systematic", "quadrant count, custom", "Ripley's K function", "Voronoi tessellation", or "nearest neighbor analysis".

Marked: Whether or not a marked point pattern analysis has been performed

Software: The software used in the counting process, indexes the lookup table "Softwares"

Stereology details record: Foreign key, referencing ID in the table "Stereology details"

Related quantification: Foreign key, referencing ID in the table "Quantitation", since many distribution analyses might be related to quantitations, although in theory they need not be.

4.8. Stereology details

Stereology details name: The name of the record, formatted as [quantitation name] or [distribution name]_stereology

Probe: The type of probe that has been used, selection of physical dissector, optical dissector, Nv Vref method, or nearest neighbor.

Identifying feature: The feature that has been used to identify events

Disector height: The height of the dissector, which corresponds to the average section thickness after processing, minus the guard height.

Area subfraction: The size of the counting frame divided by the size of the grid.

Height subfraction: Disector height divided by section thickness after processing.

Investigated sections: Mean number of investigated sections

Investigated fields: Mean number of investigated fields

Counted objects: Mean number of counted objects

Coefficient of error: Mean coefficient of error

Estimated volume: The estimated volume for the region in which objects were counted

Volume unit: The unit of the region volume, selection of nm^3, μm^3 and mm^3

5. Region records and related tables

Each morphology and quantitation is related to a region record. More than one morphology or quantitation may be related to the same region record.

5.1. Region records

Region record name: Name of the region record, formatted as [experiment name]_[ROI].

Region: The region of interest defined in HBP atlas terms, indexing the lookup table "Regions"

Secondary region: Region outside the main region of interest that might be covered

Coverage: Whether the whole region of part of region is covered

Coverage specification: Specification of the parts of the region that is not covered if coverage is not full

No original regions: The number of original regions behind the region record. If it is more than one, it means that data has been pooled to get a better translation to the relevant HBP atlas region.

Original region retained: Whether or not the original region record has been retained, i.e. not translated. This indicates that the data were already related to regions in WHS or AMBA.

Documentation score: The total documentation score, ranging from 0 to 10

Parcellation scheme: Whether or not a specific reference atlas or another parcellation scheme has been used and cited (1 point)

Atlas coordinates: Whether some form of coordinates (section level coordinates or point coordinates) are given (1 point)

Illustration: Whether or not an illustration of the region of interest is given (1 point)

Semantic description: Whether or not a semantic description of the region term is given (1 point)

Annotated images: Whether or not image documentation of the region of interest is given, either with (2 points) or without (1 point) annotations

Regional characteristics: Whether or not regional characteristics of the ROI have been documented, either cyto- or chemoarchitectonic, or electrophysiological ones (1 point)

Atlas reg: Whether or not the material has been spatially registered to a reference atlas (1 point)

Serial sections: Whether serial image documentation is given through part of (1 point) or the entire (2 points) region of interest

Collectors comment: Additional comments about the region record and / or translation process

Original framework: The original atlas framework used in the source

5.2. Original region records

One or more original region records can be related to a single record in the region records table. These original records are stored in a separate table.

Region record: Foreign key, referencing ID in region record table

Original region: The original region of interest, indexing the lookup table “Regions other”

Original coord AP min: Bregma coordinate for the most anterior point of the region of interest

Original coord AP max: Bregma coordinate for the most posterior point of the region of interest

Original coord DV min: Bregma coordinate for the most dorsal point of the region of interest

Original coord DV max: Bregma coordinate for the most ventral point of the region of interest

Original coord ML min: Bregma coordinate for the most medial point of the region of interest

Original coord ML max: Bregma coordinate for the most lateral point of the region of interest

6. Other tables

6.1. Calculations

If one or more calculations has been performed to a quantitation, this table contains information about it. It has only two fields

Quantitation ID: Foreign key, referencing ID in quantitation table

Performed calculation: The calculation performed, one of the following: SEM to SD conversion, unit conversion, data pooled from multiple specimen, data pooled from multiple regions, data pooled from multiple neurons, total number from volume and density, or density from volume and total number.

6.2. Considered papers

Table containing information about the papers identified through the literature search and considered for the database.

First author: The name of the first author, formatted as [Last name], initials

Published: The year of publication

Title: The title of the paper

Journal: The journal of the paper

Species: Species used in the study

6.3. Considered papers decisions

Table containing information about the decisions made about considered papers.

Paper: Foreign key, referencing ID in considered papers table

Decision: The decision made, i.e. whether the paper was included or excluded

Region considered: The region of interest for which the paper was considered

6.4. Lookup tables

Unless otherwise specified, lookup tables contain an incrementally increasing ID (primary key), a field with the element to be looked up, and an ontological identifier field.

6.4.1. Age categories

Lookup tables containing records of age categories.

Species: The relevant species

Age category: The age category

Ontological identifier: The preferred ontological ID found in Interlex

Description: Description of the age category limits

6.4.2. Cell phenotype categories

Lookup table containing cell phenotype categories, according to the INCF special interest group. These have no ontological identifier.

6.4.3. Cell phenotypes

This table contains information about identified cell phenotypes, and is indexed by the table "Cell type classifications".

Phenotype name: The name of the phenotype, formatted as [Phenotype category_phenotype], e.g. "Expression_dopamine".

Phenotype category: The phenotype category, as defined by the INCF special interest group. This field indexes the lookup table "cell phenotype categories", e.g. expression

Phenotype: The specific phenotype value, e.g. dopamine

Ontological identifier: The preferred ontological ID found in Interlex

6.4.4. Cellular regions

Lookup table containing cellular region terms, e.g. soma, distal dendrite, according to preferred Interlex ontological identifiers where possible.

6.4.5. Cell types

Lookup tables containing cell types, inferred from identified phenotypes in the mined studies, according to preferred Interlex ontological identifiers where possible.

6.4.6. Microscopes

Lookup tables containing microscope types, e.g. fluorescence microscope, according to BIRNLEX: ontology where possible.

6.4.7. Nomenclatures other

Lookup table containing information about non-HBP nomenclatures (atlases)

Nomenclature name: The name of the nomenclature

Version: Version of the nomenclature

Authors: Authors of the nomenclature

Species: Species the nomenclature is related to, indexes the lookup table "Species"

Strain: Strain the nomenclature is related to, indexes the lookup table "Strains"

Published: Year of atlas publication

Publication type: Type of publication, as "atlas" or "journal article"

6.4.8. Nomenclatures preferred

Lookup table containing information about HBP nomenclatures (atlases)

Nomenclature name: The name of the nomenclature

Version: Version of the nomenclature

Authors: Authors of the nomenclature

Species: Species the nomenclature is related to, indexes the lookup table “Species”

Strain: Strain the nomenclature is related to, indexes the lookup table “Strains”

Published: Year of atlas publication

Publication type: Type of publication, as “atlas” or “journal article”

6.4.9. Objects of interest

Lookup table containing objects of interest.

Object of interest: Name of the object of interest.

Ontological identifier: ID according to ILX: and GO: ontologies where possible.

6.4.10. Regions

Lookup table including regions in HBP atlases

Region name: Name of the region, formatted as [atlas abbreviation]_[region name]

Nomenclature: Foreign key, referencing ID in the “Nomenclatures table”

6.4.11. Regions other

Lookup table including regions in non-HBP atlases

Region name: Name of the region, formatted as [atlas abbreviation]_[region name]

Nomenclature: Foreign key, referencing ID in the “Nomenclatures other table”

Comments: Additional information about the structure, how it appears in the atlas, and resemblance to the same regions in other editions of the atlas

6.4.12. Region zones

Lookup table containing region zones, e.g. matrix of striatum.

Region zone: Name of the region zone

Ontological identifier: ID according to preferred Interlex ontological identifiers where possible.

6.4.13. Reporters

Lookup table containing records for each reporter (antibody or RNA probe)

Reporter name: Name of the reported formatted as [host]_anti_[target] for antibodies and [target]_[probe type] for in situ hybridization probes. Descriptive strings are added if relevant.

Reporter type: The type of reporter, selection of antibody (monoclonal, polyclonal or unspecified clonality), RNA probe, or oligonucleotide.

Reporter unique ID: Unique ID coupled to the reporter. For antibodies, this is an RRID. For in situ hybridization probes, it might be a PMID or other relevant identifier.

Target: The target of the probe

Label: The conjugate of the reporter, indexes ID in the "Reporter labels" table

Comment: Free-text field, might contain relevant information about the reporter

Origin species: The species used to produce the probe

6.4.14. Reporter labels

Lookup table containing records of tags that can be attached to a reporter, e.g. alkaline phosphatase, biotin, alexa fluor 488

Label: Name of the label

6.4.15. Reporter targets

Lookup table containing records of targets for probes, e.g. parvalbumin, substance P mRNA

Phenotype: Name of the target

6.4.16. Sectioning instruments

Lookup table including various sectioning instrument options.

Sectioning instrument name: Name of the sectioning instrument

6.4.17. Sex

Lookup table includes the options male, female according to PATO: ontology, or "both".

6.4.18. Softwares

Lookup table containing softwares

Software name: The common name of the software

Software RRID: The RRID of the software

6.4.19. Solutions

Lookup table containing chemical solutions or reagents.

Solution name: Name of the solution.

6.4.20. Source origins lookup

Lookup table containing origins for sources.

Source name: Name of the source origin.

Identifier: The RRID for the source origin, where possible.

6.4.21. Species

Lookup table includes mus musculus or rattus norvegicus according to NCBItaxon: ontology.

6.4.22. Strains

Lookup table containing strains.

Species: The species to which the strain belongs.

Strain name: Name of the strain.

Ontological identifier: ID according to BIRNLEX: and NLX: ontologies.

6.4.23. Substrains

Lookup table containing records for substrains of animals, according to BIRNLEX: ontology where possible

Species: The relevant species, indexes ID in the “Species” table

Strain name: The relevant strain, indexes ID in the “Strains” table

Substrain name: Name of the substrain. Recombinant inbred mouse strains are named as [strain 1] x [strain 2]

6.4.24. Transgenic lines

Lookup table containing records for transgenic lines of animals.

Transgenic line name: Name of the transgenic line, as reported in paper unless RRID is given, in which case the name associated with the RRID is given

Transgenic line RRID: RRID of the transgenic line

6.4.25. Visualization methods

Lookup table containing records for visualization protocols,

Protocol name: Name of the visualization protocol

Ontological identifier: ID according to BIRNLEX: or NLX_INV: ontologies where possible

6.5. Connecting tables

6.5.1. Derived data_EMdetails

This tables connects derived data records to electron microscopy details

Derived data record: Foreign key, references ID in the "Derived data records" table

EM details: Foreign key, references ID in the "Electron microscopy details" table

6.5.2. Derived data_LFMdetails

This tables connects derived data records to light / fluorescence microscopy details

Derived data record: Foreign key, references ID in the "Derived data records" table

EMdetails: Foreign key, references ID in the "Light fluorescence microscopy details" table
