# Supplementary material for: Database of literature derived cellular measurements from the murine basal ganglia
Source: Sci Data. 2020 Jul 6;7:211. doi: 10.1038/s41597-020-0550-3 (PMC7338524; doi:10.1038/s41597-020-0550-3)
Supplement: Supplementary file 2 [file 41597_2020_550_MOESM2_ESM.pdf]

Search strings used for literature search

1 Search 1 – initial overall search (Performed January 2018)

(Exp rat/ OR exp mouse/ OR mouse OR mice OR rat OR rats)

AND

(Striatum.tw,kf. OR neostriatum.tw,kf. OR “caudate putamen”.tw,kf. OR caud*putamen.tw,kf. OR accumbens.tw,kf. OR “ventral pallidum”.tw,kf. OR “substantia innominata”.tw,kf. OR innominata.tw,kf. OR paleostriatum.tw,kf. OR pallid*.tw,kf. OR “globus pallidus”.tw,kf. OR entopeduncul*.tw,kf. OR subthalam*.tw,kf. OR “substantia nigra”.tw,kf. OR “luys’ nucleus”.tw,kf. OR “luys’ body”.tw,kf. OR “corpus luisy”.tw,kf. OR “basal ganglia”.tw,kf. OR “basal nuclei”.tw,kf. OR “whole-brain”.tw,kf. OR wholebrain.tw,kf. OR “brain-wide”.tw,kf. OR brainwide.tw,kf.)

AND

(Immuno*chem* OR histochem* OR histolog* OR immunofluoresc* OR fluoresc* OR cytochem* OR “electron microscop*”.tw,kf. OR ultrastructur* OR cytoarchitect* or chemoarchitect* OR “single-cell label*”.tw,kf. OR “intracellular label*”.tw,kf. OR “in situ hybridi*”.tw,kf. OR (golgi ajd3 stain*).tw,kf.)

AND

(((number* or counted or counting or densit* or distribution* or stereolog* or quantita* or quantific*) adj5 (cell* or neuron* or synapse* or bouton* or spine*)).tw,kf.)

2 Search 2 – extended substantia nigra search

Part 1 (performed August 2018)

((Exp rat/ OR exp mouse/ OR mouse OR mice OR rat OR rats)

AND

("substantia nigra".tw,kf. OR "basal ganglia".tw,kf. OR "basal nuclei".tw,kf. OR "whole-brain".tw,kf. OR wholebrain.tw,kf. OR "brain-wide".tw,kf. OR brainwide.tw,kf.)

AND

(Immuno*chem* OR histochem* OR histolog* OR immunofluoresc* OR fluoresc* OR cytochem* OR "electron microscop*".tw,kf. OR ultrastructur* OR cytoarchitect* or chemoarchitect* OR "single-cell label*".tw,kf. OR "intracellular label*".tw,kf. OR "in situ hybridi*".tw,kf. OR (golgi ajd3 stain*).tw,kf.)

AND

(((number* or counted or counting or densit* or distribution* or stereolog* or quantita* or quantific*) adj5 (cell* or neuron* or synapse* or bouton* or spine*)).tw,kf.))

Part 2 (performed August 2018)

("substantia nigra".tw,kf. AND stereolog*))

3 Search 3 – extended striatum search

Part 1 (performed December 2018)

(Exp rat/ OR exp mouse/ OR mouse OR mice OR rat OR rats)

AND

(Striatum.tw,kf. OR neostriatum.tw,kf. OR “caudate putamen”.tw,kf. OR caud*putamen.tw,kf OR “basal ganglia”.tw,kf. OR “basal nuclei”.tw,kf. OR “whole-brain”.tw,kf. OR wholebrain.tw,kf. OR “brain-wide”.tw,kf. OR brainwide.tw,kf.)

AND

(Immuno*chem* OR histochem* OR histolog* OR immunofluoresc* OR fluoresc* OR cytochem* OR “electron microscop*”.tw,kf. OR ultrastructur* OR cytoarchitect* or chemoarchitect* OR “single-cell label*”.tw,kf. OR “intracellular label*”.tw,kf. OR “in situ hybridi*”.tw,kf. OR (golgi ajd3 stain*).tw,kf.)

AND

(((number* or counted or counting or densit* or distribution* or stereolog* or quantita* or quantific*) adj5 (cell* or neuron* or synapse* or bouton* or spine*)).tw,kf.)

Part 2 (performed January 2019)

(Striatum.tw,kf. OR neostriatum.tw,kf. OR “caudate putamen”.tw,kf. OR caud*putamen.tw,kf OR “basal ganglia”.tw,kf. OR “basal nuclei”.tw,kf. OR “whole-brain”.tw,kf. OR wholebrain.tw,kf. OR “brain-wide”.tw,kf. OR brainwide.tw,kf.)

AND

stereolog*
