# Supplementary material for: Database of literature derived cellular measurements from the murine basal ganglia
Source: Sci Data. 2020 Jul 6;7:211. doi: 10.1038/s41597-020-0550-3 (PMC7338524; doi:10.1038/s41597-020-0550-3)
Supplement: Supplementary file 3 [file 41597_2020_550_MOESM3_ESM.pdf]

Relevance	Repository name	Data	Metadata	Derived data	Anchoring possibility	New analysis required	Content description	Status
High	Neuromorpho.org					No	544 neurons from adult, control animals, from a total of 14 different laboratories. Each neuron comes with a reconstruction file, metadata, and derived data (measures)	Included
High	Mouse Brain Architecture					No	The Cell Type Portal contains an atlas of distribution of inhibitory neural cell types, for which metadata and derived data (cell counts) are available	Included
High	Allen Institute					Yes	Genome-wide in situ hybridisation data available, at least 91 data sets are potentially relevant (target structure search). Cell morphology data so far from non-BG areas only.	Not included, no derived data

Medium	GENSAT					Yes	Various gene expression data from transgenic mouse lines	Not included, no derived data
Medium	BioNumbers					No	Quantitative statements from the literature. No relevant data from basal ganglia found	Not included, no relevant data
Low	Janelia MouseLight					Yes	No reconstructions yet with soma in BG, and no derived data	Not included
High	Rodent brain workbench					Yes	IHC datasets with section series available	Not included, no derived data
High	Brainatomics					Yes	Whole brain electron microscopy datasets from rat and mouse	Not included, no derived data
