# Supplementary material for: Database of literature derived cellular measurements from the murine basal ganglia
Source: Sci Data. 2020 Jul 6;7:211. doi: 10.1038/s41597-020-0550-3 (PMC7338524; doi:10.1038/s41597-020-0550-3)
Supplement: Supplementary file 4 [file 41597_2020_550_MOESM4_ESM.pdf]

Region comparability score

<table>
<tr><th>Score</th><th colspan="2">Relationship types</th><th>Criteria</th></tr>
<tr><td>0</td><td colspan="2">Non-overlapping</td><td>The regions are non-overlapping</td></tr>
<tr><td>1</td><td colspan="2" rowspan="3">Overlapping</td><td>The regions have very little of their respective areas shared (<20%) and precise area of overlap hard to define</td></tr>
<tr><td>2</td><td>The regions have very little of respective areas shared (<20%) or precise area of overlap hard to define</td></tr>
<tr><td>3</td><td>Regions sharing more than approximately 20% of their respective areas, with non-overlapping parts easily defined</td></tr>
<tr><td>4</td><td rowspan="3">Overlapping</td><td rowspan="3">Part of
Includes</td><td>Overlapping regions that share more than approximately 40% of their respective areas, with non-overlapping parts easily defined

Part-of / includes relationships with very low coverage (>20%)</td></tr>
<tr><td>5</td><td>Overlapping regions that share more than approximately 60%, with non-overlapping parts easily defined

Part of / includes relationships with approximately 25% coverage</td></tr>
<tr><td>6</td><td>Overlapping areas that share more than 80% of their respective areas

Part of / includes relationship with approximately 50% coverage</td></tr>
<tr><td>7</td><td colspan="2">Part of / includes</td><td>Part of / includes relationships with more than approximately 70% coverage</td></tr>
<tr><td>8</td><td colspan="2" rowspan="3">Identical</td><td>Small, non-overlapping regions (>20%)</td></tr>
<tr><td>9</td><td>Small, non-overlapping regions (>10%)</td></tr>
<tr><td>10</td><td>No visible regions of non-overlap</td></tr>
</table>
