# Supplementary material for: Database of literature derived cellular measurements from the murine basal ganglia
Source: Sci Data. 2020 Jul 6;7:211. doi: 10.1038/s41597-020-0550-3 (PMC7338524; doi:10.1038/s41597-020-0550-3)
Supplement: Supplementary file 5 [file 41597_2020_550_MOESM5_ESM.pdf]

Calculation of volumetric densities

In many cases, cell counts were reported as numbers per square millimeter. To obtain a volumetric density and facilitate comparison with other density estimates, we converted these numbers. For the numbers that had not been corrected for double-counting, we applied a correction as follows:

$$N = \frac{n \times T}{T + D}$$

Where N is the cell number, n is the number of counted profiles, T is the thickness of the sections, and D is the mean diameter of the profiles. As we could not know the diameter of cells counted for each study, we used 15 µm as an approximation.

The corrected 2D cell counts were then used to calculate the volumetric density as follows:

$$vN = \frac{n}{T}$$

Where vN is the volumetric density, n is the corrected 2D cell count, and T is the thickness of the sections.
