# Supplementary material for: Database of literature derived cellular measurements from the murine basal ganglia
Source: Sci Data. 2020 Jul 6;7:211. doi: 10.1038/s41597-020-0550-3 (PMC7338524; doi:10.1038/s41597-020-0550-3)
Supplement: Supplementary file 6 [file 41597_2020_550_MOESM6_ESM.pdf]

Database entry

Generally, if ranges were given for a parameter where our database stored only a single value (e.g. time variables, number of animals), the mean of the upper and lower bound was entered. When collecting information about tissue treatment procedures, we sometimes encountered papers where broad time ranges were given for time sensitive incubations (e.g. saying that "DAB-staining was carried out for 5-30 minutes"). In these cases, we did not enter any information about time in the database so as not to give a false impression of having precise time information. For ranges of number of animals, the lower bound was entered. If a range of coefficient of errors was given across groups without indication of which value belonged to which group, the higher bound was used.

If it was stated that stereology had been performed using a certain software or probe, it was assumed that subsampling had been done in an unbiased way and that the final estimation basis was a stereological formula, since these are inherent parts of such software or procedures. Systematic random sampling of sections was not assumed unless specifically stated.

Two criteria had to be met to assume region coverage and / or specificity. First, documentation should be provided using either: 1) images or illustrations throughout the region; 2) a range of section coordinates covering the region; or 3) a reference to the region in a specific parcellation scheme, with a statement that the entire region was sampled. Secondly, to assume whole region coverage, the documented region had to have an 'identical' or 'includes' relationship with a region in an HBP atlas; to assume specificity, the region described in the paper needed to have an 'identical' or 'part of' relationship with a region in an HBP atlas. To assume both specificity and coverage, the relationship needed to be of the 'identical' type.

In some cases, calculations were performed to standardize the data. This mainly involved converting standard errors to standard deviations and unit conversions (e.g. converting cells per 1000 µm2 to cells per µm2). If numbers from several control groups of similar sex, species, strain, age and treatment were given, the results were pooled and entered under a single experiment. In some cases, data from several regions of interest were pooled, but only when this was consider to clearly increase the accuracy of region translation (see above). In all cases where calculations were performed, this was clearly indicated in the database (see description of "Calculations" table in Supplementary file 1), and an excel sheet was made to document the applied calculations.

The term "overnight" was interpreted as 12 hours

The term "room temperature" was interpreted 21 degrees Celsius

When data for several subdivisions of a region was given (e.g. from dorsolateral and ventromedial caudoputamen), both numbers were entered with the same region record (for the overall caudoputamen). When numbers for both the region as a whole and for specific subregions were given, both were entered. The information about the specific subdivision was then given in the "region zone"

field of the quantitation record. Exceptions were made when the subdivisions were deemed to add up to a well-defined atlas region. In these cases, numbers were added up (total numbers) or averaged (densities) and this was indicated in the Calculations table.

When data from both hemispheres were given separately for animals that had undergone a sham intracranial procedure, the contralateral numbers were used.

When data from both hemispheres were given separately for animals with no intracranial procedure, both numbers were entered separately
