# Supplementary material for: Database of literature derived cellular measurements from the murine basal ganglia
Source: Sci Data. 2020 Jul 6;7:211. doi: 10.1038/s41597-020-0550-3 (PMC7338524; doi:10.1038/s41597-020-0550-3)
Supplement: Supplementary file 7 — Supplementary figure 1 [file 41597_2020_550_MOESM7_ESM.pdf]

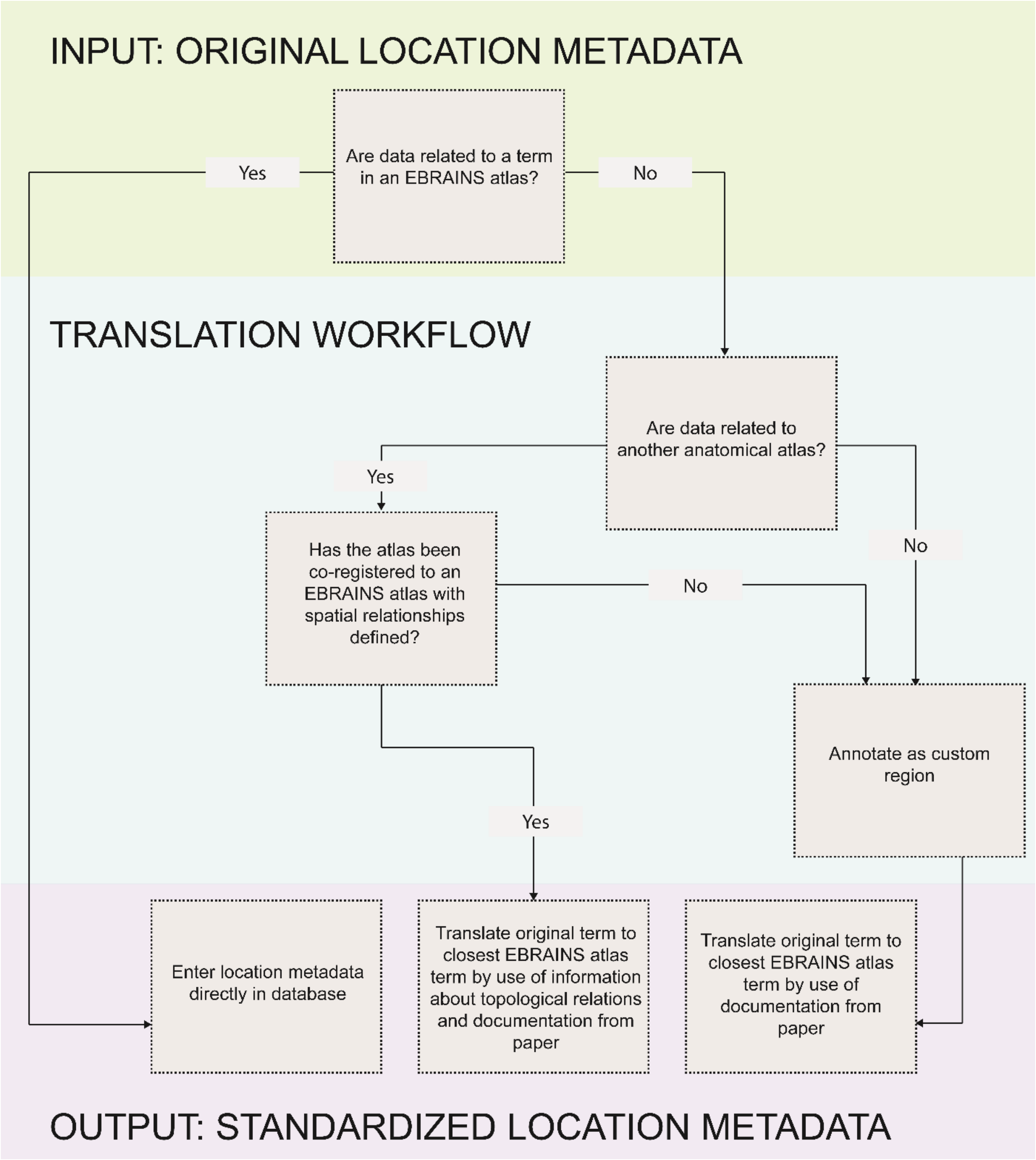


**Supplementary Figure 1. Atlas region translation workflow**

Figure illustrating the workflow for semantic translation of atlas region terms. Original location metadata found in papers are taken through a translation workflow to obtain standardised location metadata. Location metadata that are consistent with the nomenclature of any of the EBRAINS atlases (Waxholm Space atlas of the rat brain[1–3] and Allen mouse brain Common Coordinate Framework[4]) are entered directly in the database. If location metadata are consistent with terms in another anatomical atlas, they can be translated

to EBRAINS atlas terms based on the relationships between regions in the relevant atlases[5]. If location metadata are not consistent with any anatomical atlas, the terms are annotated as “custom”, and translations are made based on the documentation provided in the paper. Location metadata that are consistent with an atlas for which spatial relationships are not available in the EBRAINS dataset are treated as custom regions.


1. Papp, E., Leergaard, T. T. B., Calabrese, E., Johnson, G. A. G. & Bjaalie, J. G. J. Waxholm Space atlas of the Sprague Dawley rat brain. *Neuroimage* **97**, 374–386 (2014).
2. Kjonigsen, L., Lillehaug, S., Bjaalie, J., Witter, M. & Leergaard, T. Waxholm Space atlas of the rat brain hippocampal region: Three-dimensional delineations based on magnetic resonance and diffusion tensor imaging. *Neuroimage* **108**, 441–449 (2015).
3. Osen, K., Imad, J., Wennberg, A., Papp, E. & Leergaard, T. Waxholm Space atlas of the rat brain auditory system: Three-dimensional delineations based on structural and diffusion tensor magnetic resonance imaging. *Neuroimage* **199**, 38–56 (2019).
4. Oh, S. *et al.* A mesoscale connectome of the mouse brain. *Nature* **508**, 207–214 (2014).
5. Bjerke, I., Puchades, M., Bjaalie, J. & Leergaard, T. Comparability of basal ganglia delineations across different mouse brain atlases. (2019) doi:https://doi.org/10.25493/MWAS-3S6.
